# New AMS ^14^C dates track the arrival and spread of broomcorn millet cultivation and agricultural change in prehistoric Europe

**DOI:** 10.1038/s41598-020-70495-z

**Published:** 2020-08-13

**Authors:** Dragana Filipović, John Meadows, Marta Dal Corso, Wiebke Kirleis, Almuth Alsleben, Örni Akeret, Felix Bittmann, Giovanna Bosi, Beatrice Ciută, Dagmar Dreslerová, Henrike Effenberger, Ferenc Gyulai, Andreas G. Heiss, Monika Hellmund, Susanne Jahns, Thorsten Jakobitsch, Magda Kapcia, Stefanie Klooß, Marianne Kohler-Schneider, Helmut Kroll, Przemysław Makarowicz, Elena Marinova, Tanja Märkle, Aleksandar Medović, Anna Maria Mercuri, Aldona Mueller-Bieniek, Renato Nisbet, Galina Pashkevich, Renata Perego, Petr Pokorný, Łukasz Pospieszny, Marcin Przybyła, Kelly Reed, Joanna Rennwanz, Hans-Peter Stika, Astrid Stobbe, Tjaša Tolar, Krystyna Wasylikowa, Julian Wiethold, Tanja Zerl

**Affiliations:** 1 Institute for Prehistoric and Protohistoric Archaeology, Kiel University, Johanna-Mestorf-Str. 2-6, 24118 Kiel, Germany; 2 Centre for Baltic and Scandinavian Archaeology (ZBSA), Schleswig-Holstein State Museums Foundation, Schloss Gottorf, 24837 Schleswig, Germany; 3 Leibniz-Laboratory for AMS Dating and Stable Isotope Research, Kiel University, Max-Eyth-Str. 11-13, 24118 Kiel, Germany; 4 Akademie der Wissenschaften und der Literatur, Geschwister-Scholl-Straße 2, 55131 Mainz, Germany; 5 Integrative Prähistorische und Naturwissenschaftliche Archäologie IPNA, Basel University, Spalenring 145, 4055 Basel, Switzerland; 6 Lower Saxony Institute for Historical Coastal Research, Viktoriastraße 26/28, 26382 Wilhelmshaven, Germany; 7 Dipartimento di Scienze della Vita, Università degli Studi di Modena e Reggio Emilia, Via Giuseppe Campi 287, 41125 Modena, Italy; 8 Facultatea de Istorie şi Filologie, Universitatea “1 Decembrie 1918” Alba Iulia, Strada Unirii 15-17, 510009 Alba Iulia, Romania; 9 Institute of Archaeology of the Czech Academy of Sciences, Prague, Letenská 4, 118 01 Praha 1, Czech Republic; 10 Effenberger Archäobotanik, 21423 Drage, Germany; 11 Department of Nature Conservation and Landscape Ecology, Szent István University, Páter Károly utca 1, Gödöllő, 2103 Hungary; 12 Austrian Archaeological Institute (ÖAI), Austrian Academy of Sciences (ÖAW), Franz Klein-Gasse 1, 1190 Vienna, Austria; 13 Landesamt für Denkmalpflege und Archäologie Sachsen-Anhalt—Landesmuseum für Vorgeschichte, Richard-Wagner-Str. 9, 06114 Halle (Saale), Germany; 14 Brandenburgisches Landesamt für Denkmalpflege und Archäologisches Landesmuseum Ortsteil Wünsdorf, Wünsdorfer Platz 4-5, 15806 Zossen, Germany; 15 Władysław Szafer Institute of Botany, Polish Academy of Sciences, Lubicz 46, 31-512 Kraków, Poland; 16 Archäologisches Landesamt Schleswig-Holstein, Brockdorff-Rantzau-Straße 70, 24837 Schleswig, Germany; 17 Department für Integrative Biologie, Universität für Bodenkultur, Gregor-Mendel-Straße 33, 1180 Vienna, Austria; 18 Independent Researcher, Projensdorfer Str. 195, 24106 Kiel, Germany; 19 Faculty of Archaeology, Adam Mickiewicz University, Uniwersytetu Poznańskiego 7, 61-614 Poznań, Poland; 20 Landesamt für Denkmalpflege am Regierungspräsidium Stuttgart, Fischersteig 9, 78343 Gaienhofen-Hemmenhofen, Germany; 21 Museum of Vojvodina, Dunavska 35-37, 21101 Novi Sad, Serbia; 22 Dipartimento di Studi sull’Asia e sull’Africa Mediterranea, Università Ca’ Foscari, Dorsoduro 3462, 30123 Venezia, Italy; 23 National Museum of Natural Sciences of the National Academy of Sciences in Ukraine, Bul. Bohdan Khmelnitsky 15, Kyiv, 01030 Ukraine; 24 Laboratory of Palynology and Palaeoecology CNR IGAG, Piazza della Scienza 1, 20126 Milan, Italy; 25 Centre for Theoretical Study, Charles University Prague and Czech Academy of Sciences, Jilská 1, 110 00 Prague 1, Czech Republic; 26 Department of Anthropology and Archaeology, University of Bristol, 43 Woodland Road, Bristol, BS8 1UU UK; 27 Institute of Archaeology and Ethnology, Polish Academy of Sciences, Rubież 46, 61-612 Poznań, Poland; 28 Institute of Archaeology, Jagiellonian University, Ul. Gołębia 11, 31-007 Kraków, Poland; 29 Oxford Martin School, University of Oxford, 34 Broad Street, Oxford, OX1 3BD UK; 30 Department of Molecular Botany, Institute of Biology, University of Hohenheim, Garbenstraße 30, 70599 Stuttgart, Germany; 31 Institute of Archaeological Sciences, Johann Wolfgang Goethe University, Norbert-Wollheim-Platz 1, 60629 Frankfurt am Main, Germany; 32 ZRC SAZU, Institute of Archaeology, Novi trg 2, 1000 Ljubljana, Slovenia; 33 Institut national de recherches archéologiques préventives (Inrap), Direction régionale Grand Est, 12, rue de Méric, CS 80005, 57063 Metz cedex 2, France; 34 UMR 6298, ArTeHiS Dijon, Dijon, France; 35 Institute for Pre- and Protohistory, University of Köln, Weyertal 125, 50923 Köln, Germany

**Keywords:** Environmental social sciences, Plant sciences

## Abstract

Broomcorn millet (*Panicum miliaceum* L.) is not one of the founder crops domesticated in Southwest Asia in the early Holocene, but was domesticated in northeast China by 6000 bc. In Europe, millet was reported in Early Neolithic contexts formed by 6000 bc, but recent radiocarbon dating of a dozen 'early' grains cast doubt on these claims. Archaeobotanical evidence reveals that millet was common in Europe from the 2nd millennium bc, when major societal and economic transformations took place in the Bronze Age. We conducted an extensive programme of AMS-dating of charred broomcorn millet grains from 75 prehistoric sites in Europe. Our Bayesian model reveals that millet cultivation began in Europe at the earliest during the sixteenth century bc, and spread rapidly during the fifteenth/fourteenth centuries bc. Broomcorn millet succeeds in exceptionally wide range of growing conditions and completes its lifecycle in less than three summer months. Offering an additional harvest and thus surplus food/fodder, it likely was a transformative innovation in European prehistoric agriculture previously based mainly on (winter) cropping of wheat and barley. We provide a new, high-resolution chronological framework for this key agricultural development that likely contributed to far-reaching changes in lifestyle in late 2nd millennium bc Europe.

## Introduction

Broomcorn millet (*Panicum miliaceum* L., Poaceae family) is a resilient, fast-growing, water-efficient, drought-tolerant plant that copes well on poor soils, is successful in both low and high altitudes and across a wide latitudinal range, produces many nutrient-rich grains and abundant biomass, and serves as food, fodder, raw material and perhaps as symbol in rituals^1,2^ [131, 153],^3–7^. The agrarian methods used in growing and processing of wheat and barley are applicable to this crop^8–10^, save for, perhaps, more intensive weeding required by millet, especially early in the growing cycle cf.^11^. In particular, the short growing season of up to three months, which can be completed during the summer, makes broomcorn millet highly attractive to different agrarian systems. In cultivation regimes focused on winter crops, it offers an additional harvest, increasing annual grain production and preventing famine in years when winter crops fail. It is also aptly suited to economic systems where cultivation is practiced only during a short time-window, such as may have been the case with pastoral groups of Bronze Age Central Asia^12^, or where the availability of cropping areas was seasonally restricted (e.g. in floodplains^13^). It offers lush and abundant forage for animals, indispensable in the dry season. In cuisine, it adds to taste and texture, and diversifies the food options^7^. These qualities appeal to modern-day millet farmers in Europe^9^; they were at least part of the reason why broomcorn millet became a staple crop in Asia and Europe. In some regions nowadays, broomcorn millet is a cornerstone of food economy and a guarantee of food security^14^.

## Early millet in Europe: the question of ‘when?'

Broomcorn millet grains are common at later prehistoric sites in Europe^15–18^. These finds testify to the far-reaching dispersal of the crop that was first domesticated in modern-day China. Its spread and adoption across central and western Asia and Europe is one of the most clear-cut examples of 'food globalisation' or ‘trans-Eurasian exchange’ of technologies and inventions between the distant parts of the "Eurasian dyad"^19–24^. The earliest recorded finds of broomcorn millet in China in the form of charred grains derived from Early Neolithic sites in the north/north-east of the country and have been dated to the early-mid 6th millennium bc^4,25,26^. In Europe, archaeobotanical finds of broomcorn millet were previously reported for Neolithic sites whose occupation started as early as the late 7th millennium bc (e.g. in the southern and eastern Balkans), as well as a number of later pre-2nd millennium bc sites^17^. However, radiocarbon dating of ten charred grains from Neolithic (6–5th millennium bc) layers demonstrated their (much) later date, not earlier than the very end of the seventeenth century bc^27^. This discovery cast doubt on any reported 'Neolithic' broomcorn millet in Europe, and hinted that the east-to-west transfer of this crop might have taken place later than thought. The earliest so far directly dated broomcorn millet grains in Central Asia date to the mid–late 3rd millennium cal bc (Begash, Kazakhstan: 2470–2190 cal bc at 94.7% confidence (Beta-266458, 3840 ± 40 BP^28,29^); Adji Kui 1, Turkmenistan: 2210–1960 cal bc at 94.0% confidence (3708 ± 45 BP, no laboratory code^30^); southeast from this region, in the Kashmir Valley, millet from the site of Pethpuran Teng, Kashmir, is equally early (2580–2450 cal bc at 92.3% confidence [D-AMS 033182, 3981 ± 34 BP]^31^). Nevertheless, persistent reports of European finds of pre-2nd millennium bc broomcorn millet grains have kept open the question of when broomcorn millet arrived in Europe.

**Figure 1:**
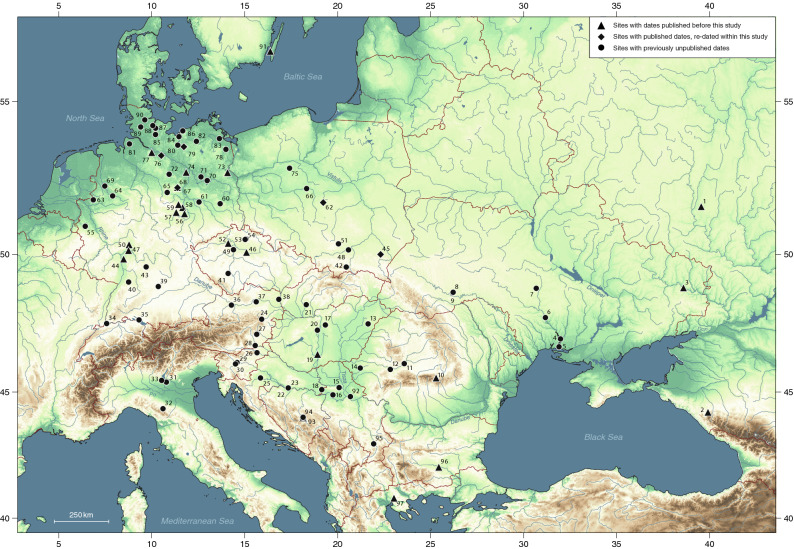
The location of sites in Europe with published and new broomcorn millet dates: (1) Rykan; (2) Guamsky Grot; (3) Zanovskoe; (4) Dikiy Sad; (5) Olbia; (6) Vinogradnyi Sad; (7) Maidanetske; (8) Zalissya; (9) Ivane-Puste; (10) Măgura-Buduiasca; (11) Teleac; (12) Miercurea-Sibiului; (13) Debrecen; (14) Cornești; (15) Kalakača; (16) Gomolava; (17) Pécel; (18) Bosut; (19) Fajsz; (20) Százhalombatta; (21) Vráble; (22) Crišnjevi; (23) Oštrovi; (24) Gasteil; (25) Lasinja; (26) Orehova Vas; (27) Neudorf; (28) Retznei; (29) Dragomelj; (30) Tribuna; (31) Custoza; (32) Santa Giulia; (33) Lavagnone; (34) Binningen; (35) Hagnau; (36) Ansfelden; (37) Meidling; (38) Stillfried; (39) Ipf; (40) Knittlingen; (41) Pisek-Sever; (42) Maszkowice; (43) Königshofen; (44) Goddelau; (45) Lipnik; (46) Velim; (47) Fechenheim; (48) Witów; (49) Roztoky; (50) Bruchenbrücken; (51) Miechów; (52) Zahájí; (53) Valečov; (54) Soví převis; (55) Jülich-Güsten; (56) Schafstädt; (57) Niederröblingen; (58) Bösenburg; (59) Quenstedt; (60) Großbahren; (61) Radis; (62) Lutomiersk; (63) Borken; (64) Warendorf; (65) Watenstedt; (66) Szczepidło; (67) Hundisburg; (68) Olbetal; (69) Altenrheine; (70) Potsdam; (71) Möthlow; (72) Lüdelsen; (73) Rathsdorf; (74) Walsleben; (75) Smuszewo; (76) Rullstorf; (77) Hittfeld; (78) Pasewalk; (79) Badegow; (80) Schwerin; (81) Flögeln; (82) Vogelsang; (83) Butzow; (84) Wismar; (85) Wahlstedt; (86) Zweedorf; (87) Depenau; (88) Flintbek; (89) Borgstedt; (90) Brekendorf; (91) Risinge; (92) Starčevo; (93) Okolište; (94) Donje Moštre; (95) Hisar; (96) Yabalkovo; (97) Assiros.[Map generated using the software: QGIS 3.10.5—A Coruña (https://qgis.org); copyright by OpenStreetMap contributors, terrestris GmbH & Co KG (https://www.terrestris.de), and Natural Earth Data (https://www.naturalearthdata.com)].

**Figure 2:**
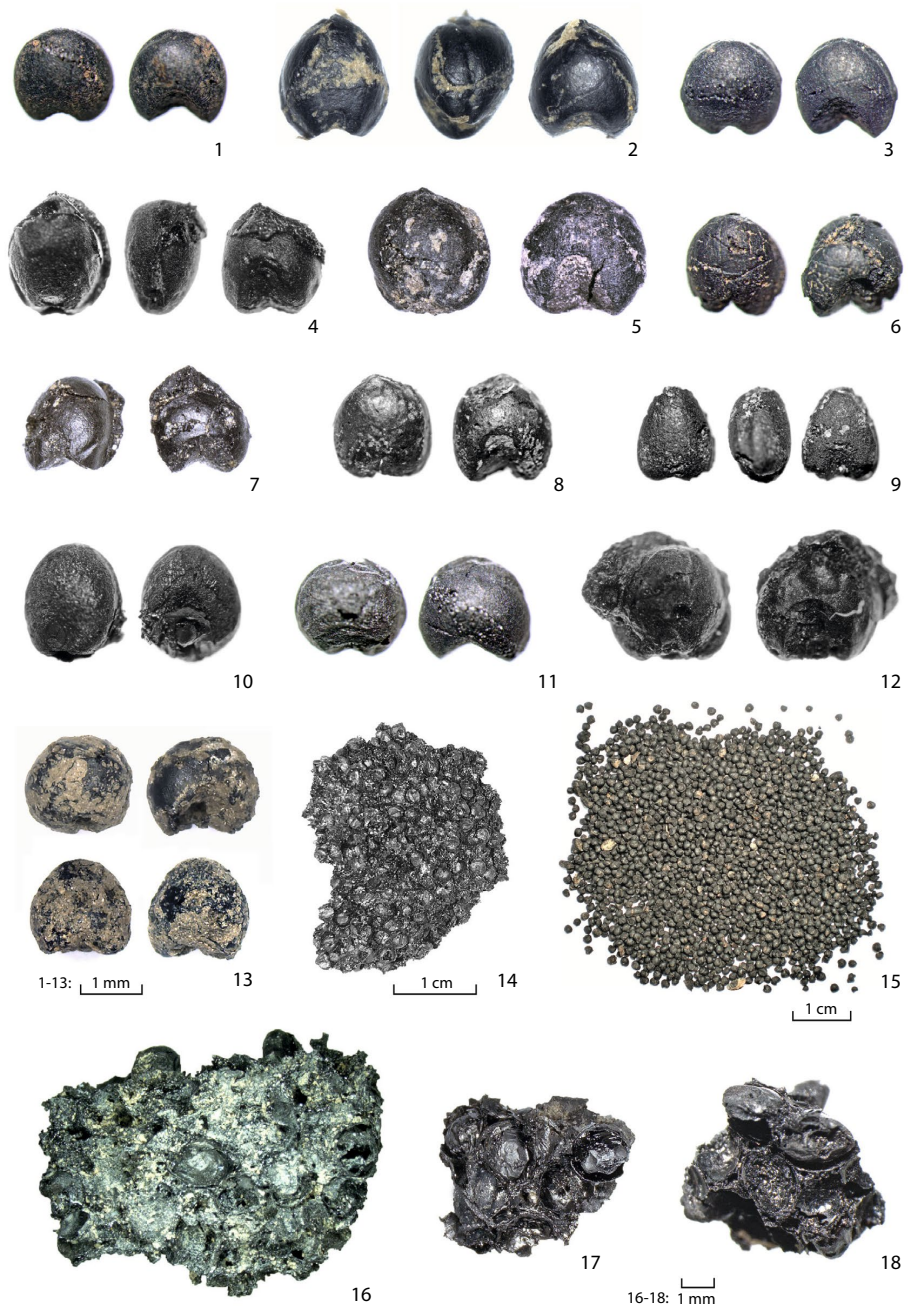
Examples of the dated loose and fused broomcorn millet grains from sites in Europe: (1) Altenrheine; (2) Vráble; (3) Pécel; (4) Teleac; (5) Ipf; (6) Binningen; (7) Königshofen; (8) Oštrovi; (9) Maszkowice; (10) Lavagnone; (11) Santa Giulia; (12) Soví převis; (13) Meidling-im-Thale; (14) Stillfried; (15) Wismar; (16) Custoza; (17) Hagnau; (18) Kalakača.

The precise chronology and character of the spread of broomcorn millet is the key to understanding the processes by which it entered Europe and the mechanisms for its uptake and final incorporation within food production, diet, cuisine, and the wider socio-economy. We therefore conducted a large radiocarbon-dating programme, targeting 'early' millet finds in eastern, central and northern Europe, and obtaining over 100 AMS-dates on charred grains from c. 70 sites (Figs. 1, 2). This paper presents the results and, in combination with the published data, uses them to address the questions of *When* and *Where* broomcorn millet arrived in several large regions of Europe, which we refer to as North Pontic, Carpathian Basin, Po Basin, central Europe and North-central Europe. We do this by (a) modelling the spread of the crop within the selected parts of Europe using a Bayesian approach, and (b) weaving together radiocarbon dating evidence and relevant archaeobotanical and stable isotope data.

## Results

### New AMS measurements

Given the size of charred broomcorn millet grains (e.g. 1.7–2.0 mm in length, 1.4–1.7 mm in breadth^2^ [131]; 0.78–1.53 mg per grain^32^ [Table 31]), the dry mass prior to chemical pretreatment was sometimes very low (0.4–0.9 mg for 19 specimens, Supplementary Dataset). Extraction yields varied, due to differences in grain preservation conditions. We disregarded results from samples yielding < 100 μg (0.1 mg) of carbon, as their ^14^C ages are too imprecise to be informative. For most samples, the AMS δ^13^C value (which is used to correct the measured ^14^C concentration for fractionation) is lower than the expected δ^13^C value for charred broomcorn millet (c. − 11.1 ± 0.5‰^33^ [319]). The low AMS δ^13^C values may be partly due to fractionation during sample preparation (oxidation and graphitisation), but is more likely due to fractionation in the AMS (most probably at the ion source), which is pronounced with small graphite targets (Goslar, T. personal communication via email, 4 May 2020). Indeed, an exponential regression of AMS δ^13^C against graphite weight predicts an average δ^13^C value of c. − 10‰ for normal-size targets (1 mg), which falls in the range expected for C_4_ plants, with increasing fractionation (more negative δ^13^C values) as the dating targets get smaller (Supplementary Fig. 1).

### Radiocarbon dates on broomcorn millet grains in Europe: the dataset

Table 1 summarizes the available AMS-dates on broomcorn millet in the study region and in South-east Europe. They include 52 legacy dates (31%; including 17 previously unpublished dates) and 116 dates (69%) from this study (see Supplementary Dataset). One is on abundant uncharred broomcorn millet remains preserved in a bog at the site of Zahájí (Czech Republic)^34^; all others are on charred grain. The previously obtained dates were examined for δ^13^C values, sample weight and the material submitted for dating, as recorded in the laboratory database or published report. We established that seven published dates from seven sites and three unpublished dates from two sites were not from, or not only from, broomcorn millet grains (Supplementary Dataset). The combined remains of broomcorn millet and free-threshing wheat dated at Begash in Central Asia were clearly from the same context^28^ [1004]. Although there are similar examples in Europe, we disregarded such cases and focused on dates exclusively from broomcorn millet grains.

**Table 1:** The number of published, unpublished and newly produced AMS-dates reported as obtained on broomcorn millet from Europe.

Status	Number of dated sites	Number of AMS dates
Published before this study	27	35
Unpublished dates included in this study	6	17
Dates produced within this study	69^a^	116
Total	97	168

Our analysis showed that not all of the 'millet-dates' were obtained (exclusively on) broomcorn millet (see remarks in Supplementary Dataset).

^a^Includes five sites with published dates, re-dated within this study.

### Modelling the chronology of spread of broomcorn millet in Europe

Our preferred Bayesian chronological model (Supplementary Fig. 2; Supplementary Model Code; Supplementary Text 1) uses 136 dates from 80 sites, out of the 153 apparently accurate dates available on broomcorn millet from 90 sites. Nineteen dates clearly falling after 1 cal bc (six published dates from four sites and 13 fresh dates from eight sites) were omitted. Multiple iterations of the preferred model revealed two early ‘misfits’, from Lavagnone in northern Italy and Ipf in southern Germany (Supplementary Dataset). Both results were too early for their positions in the overall model (individual index of agreement ≪ 60^35^) and inconsistent with other millet ^14^C dates from the same sites and the region. These results were excluded from the preferred model.

**Figure 3:**
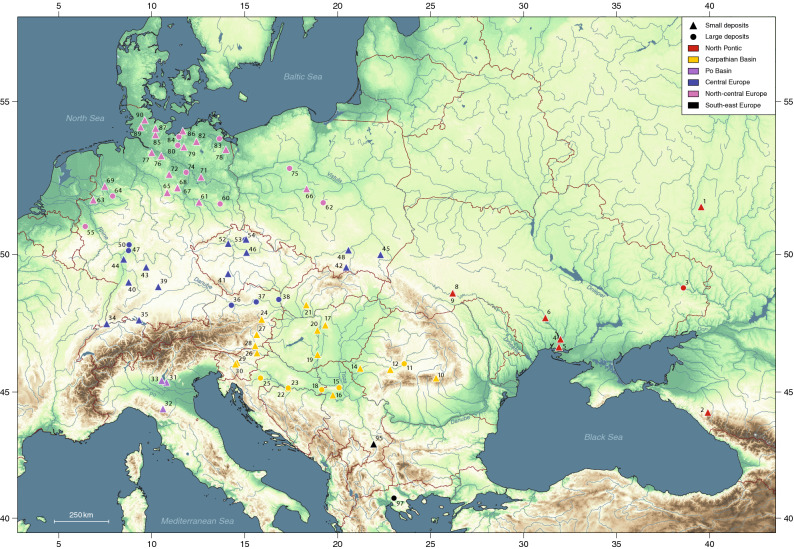
Location of the 80 sites, with small and/or large millet deposits, included in the model and assigned to broad geographical regions within which the results are discussed; see Fig. 1 caption for site names (site #4, Dikiy Sad, which yielded both small and large millet deposit, is here classified as large).[Map generated using the software: QGIS 3.10.5—A Coruña (https://qgis.org); copyright by OpenStreetMap contributors, terrestris GmbH & Co KG (https://www.terrestris.de), and Natural Earth Data (https://www.naturalearthdata.com)].

We subdivided the study region (Fig. 3) into sub-regions, based on the comparison of model outputs from various permutations of the dataset (Supplementary Text 2; Supplementary Fig. 4). The calibrated ^14^C results provide *termini ante quos* for the earliest occurrence of millet in a subregion, whose date can be estimated from the scatter of sample dates. Given the uneven spatial coverage in the data, which is an inevitable consequence of research history, we cannot standardize the number of sites and samples in each region (Table 2). The uncertainty in start-date varies regionally, depending primarily on data density (the number of samples, the number of dated sites and the proportion of these dating close to the beginning of millet cultivation), but also on measurement precision and the shape of the calibration curve (we expect the next iteration of the international ^14^C calibration curve, due to be released in 2020, will shift the oldest dates in our study slightly later, based on the new data^36,37^). Thus the estimated date of the start of broomcorn millet cultivation is fairly precise in three regions (Carpathian Basin, central Europe, North-central Europe), each represented by over 30 samples from at least 19 sites (Supplementary Dataset). The Po Basin includes only three dated sites, but their six dates are tightly clustered, whereas only one of the seven sites included in the North Pontic region is particularly early, and the others span a wide date range. The South-east Europe region includes only three dates from two sites, and the estimated start date is correspondingly vague.

**Table 2:** Estimated dates for the start of millet cultivation in each subregion, given by the Bayesian chronological model (Supplementary Text 1, 2) and shown in Figs. 4, 5.

Subregion	Number of sites in the model	Number of AMS-dates in the model		68% Probability	95% Probability
		On small deposits	On large deposits		
North Pontic	8	8	6	*1650–1500* *cal*bc	*1780–1450* *cal*bc
Carpathian Basin	20	33	7	*1480–1420* *cal*bc	*1510–1410* *cal*bc
Po Basin	3	5	1	*1500–1430* *cal*bc	*1570–1410 cal*bc
central Europe	19	22	9	*1470–1400* *cal*bc	*1490–1330* *cal*bc
North-central Europe	28	25	15	*1230–1160* *cal*bc	*1260–1140* *cal*bc
South-east Europe	2	0	3	*1500–1290* *cal*bc	*1840–1270* *cal*bc

**Figure 4:**
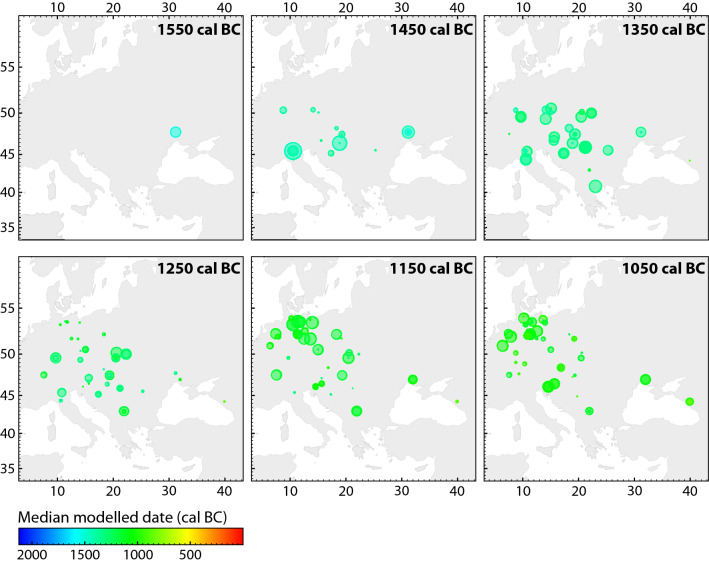
Time-slices of modelled dates of broomcorn millet grains, at 100-year intervals. Symbol size corresponds to the relative probability that a sample is of the date shown. Symbol colour corresponds to the median date of the sample (see legend).

Figure 4 maps the modelled dates, in 100-year time-slices. The only millet in Europe directly dated to the sixteenth century cal bc comes from Vinogradnyi Sad in southwestern Ukraine. By the mid-fifteenth century bc, however, broomcorn millet was apparently present in the Middle Danube region—in the territory of modern-day Romania (Corneşti*-*Iarcuri), Hungary (Fajsz 18) and perhaps Croatia (Mačkovac-Crišnjevi)—and in northern Italy (Lavagnone). By the mid-fourteenth century bc, it spread across central Europe and as far south as the Aegean (Assiros in Greece). There is little evidence that millet spread north of Bohemia during the thirteenth century bc, but by the mid-twelfth century bc it was common in northern Germany and Poland. By this date, broomcorn millet was probably cultivated throughout the study region. The ostensible gap in the western Balkans reflects the state of research, as few sites here have been analysed archaeobotanically. That broomcorn millet was present here at least from the mid-thirteenth century bc onwards is demonstrated by dates on the grains from Hisar (southern Serbia). Eastern and southern parts of the peninsula (Albania, North Macedonia, Bulgaria, Greece) were not included in this dating programme as the material from some of these regions is a part of a different study. However, the only so-far published direct-dated millet find from Greece—that from Assiros—is included in the model. A charred food crust in a pot from the Late Bronze Age site of Kush Kaya in southern Thrace (Bulgaria) contained fragments of broomcorn millet grain, along with barley and flax; the crust was directly dated to the mid-fourteenth century bc^38^ (the date is not used in our model).

Previous radiocarbon dating of broomcorn millet called into question the reported pre-2nd millennium bc occurrences of the crop in Europe^27^. Our study shows that it is highly unlikely that broomcorn millet reached central Europe prior to the mid-2nd millennium bc.

### The age of ‘small’ and ‘large’ broomcorn millet deposits

Archaeobotanical assessments of the early distribution of broomcorn millet in Europe suggested its widespread presence prior to and during the Early and Middle Bronze Age (before mid-2nd millennium bc), but as a *minor* crop^18,39,40^. This was inferred from the overall number per site of millet grains or of contexts in which they were found. The finds attributed to the Neolithic, Copper and Early Bronze Ages usually occur in the form of one to several grains per deposit and were interpreted as indicating that broomcorn millet initially was a tolerated accidental inclusion in the harvests of major crops. In contrast, dense, concentrated deposits (with grains often fused into ‘porridge’) of the Middle and Late Bronze Age were taken as reflecting a higher degree of use and/or greater importance of this crop relative to others. It was, therefore, concluded that “…the transformation of millets from minor to major (or even main) crops took place in the transition from Middle to Late Bronze Age in Europe”^18^ [361].

Based on the published archaeobotanical records of broomcorn millet finds in Europe, our expectation was that the number of obvious large deposits per subregion will be small for the earlier part of the covered period (Neolithic-Copper Age), moreover—that there may be none. Rather than seeing this as a 'proof' that millet first appeared in the respective subregion as a contaminant of other crops, we acknowledged that this can be a result of taphonomy (such as e.g. the possibility that small deposits represent re-deposited material from larger deposits) and the state of research. The radiocarbon dates presented here allow us to evaluate this notion at a macro-regional (sub-continental) level. The chronological distribution of small and large deposits of *dated* pre-Roman millet grains included in the model is summarised in Supplementary Fig. 3, their number in Table 2, and their geographical distribution in Fig. 3. Supplementary Fig. 3 shows that the overall temporal distribution of small deposits is skewed towards early dates, as predicted. In all but one subregion (North Pontic, with lowest data density), at least one of the early dates comes from a large deposit. Therefore, we would suggest that *among the dated deposits* in the study region, there is no real difference in the temporal distribution of large and small millet deposits, and that our results therefore do not support the impression that millet began as a minor crop and only became a major (or the main) crop later. Earlier deposits are not necessarily small, and there are both early and late large deposits. Small deposits also occur throughout the period of interest. There is a higher relative incidence of small deposits in the late 2nd–early 1st millennium cal bc, but many of these samples are clearly intrusive (in Neolithic contexts), and this pattern does not necessarily mean that the importance of millet declined over time.

The northern German dataset forms a useful case-study because of its size and good regional coverage. Here, the earliest large deposits of broomcorn millet grain are as old as the first small deposits (isolated grains). This suggests that broomcorn millet was established and cultivated on a larger scale and more evenly from the outset. Regular cultivation of a crop should produce both large and small deposits, but the visibility of both will depend on their preservation and discovery. Regional differences in fieldwork methods and sampling priorities may also produce misleading patterns.

This study confirms the previous finding from radiocarbon dating of broomcorn millet grains—that small deposits can be (much) younger than the archaeological layers in which they are found. Our programme also included sites where broomcorn millet was more frequent than just one or few grains per context (see Supplementary Dataset). Even in some of these cases, however, archaeological dating proved to be misleading. This particularly concerns the much-cited Late Neolithic (late 4th/early 3rd millennium bc) site of Meidling-im-Thale (Kleiner Anzingerberg), where 83 millet grains were found in several features within a burnt house^41^ [67], but radiocarbon dating placed the grains from four features no earlier than the eighth century bc. Likewise, over 670 millet grains were extracted from 31 samples from Late Neolithic and Copper Age layers (5th–4th millennium bc) at Gomolava^42^ [Table 2]. Three of these ‘millet-rich’ samples were dated directly, and none is older than tenth/ninth century bc. In both cases, the number of broomcorn millet grains per site was relatively high, but the millet-containing deposits were of low density (one or few millet grains per L/kg of soil).

## Discussion

### Routes and timing of broomcorn millet diffusion in Europe

The results of the radiocarbon dating program are here discussed in conjunction with the selected archaeobotanical (including grain imprints) and stable isotopic evidence. Adding a C_4_ plant, such as broomcorn millet, to diets otherwise based on plants using the C_3_ photosynthetic pathway increases δ^13^C values of bones and teeth, because δ^13^C values of C_4_ plants are typically 10–15‰ higher than those of C_3_ plants. There are wild C_4_ plants in Asia and some also occur in Europe (e.g. certain chenopods and grasses, especially halophytes) in salt marshes or in steppe environments, where they can be seasonally more abundant than C_3_ plants^42^, but higher δ^13^C values in prehistoric humans and domestic animals in Europe are more plausibly related to regular consumption of domesticated millets than to increasing amounts of wild C_4_ plants in graze or fodder^43,44^. Human bone collagen δ^13^C values are especially sensitive to the quantity of protein-rich freshwater or marine foods consumed, as well as the consumption of meat and milk from domestic herbivores which consumed C_4_ plants, whereas tooth enamel or bone apatite δ^13^C values reflect δ^13^C values in the overall diet, including carbohydrates. Appropriate threshold δ^13^C values identifying the input of C_4_ foodstuffs will therefore depend on the tissues analysed, the carbon contribution of aquatic species, and the δ^13^C values of local C_3_ and C_4_ plants. In Europe, for fully terrestrial diets, human bone collagen δ^13^C values ≥ c. − 18‰ may signify inclusion of C_4_ plants ("mixed C_3_/C_4_ diet"); δ^13^C values ≥ c. − 12‰ reflect a "predominantly C_4_ diet"^45,46^.

**Figure 5:**
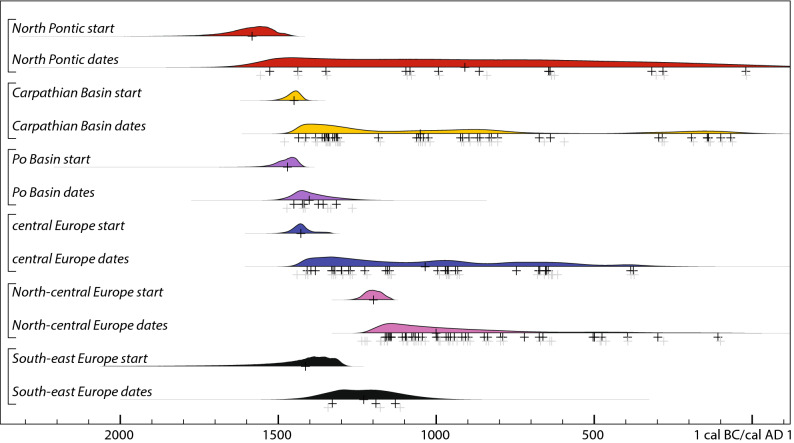
Modelled start date of millet cultivation (OxCal function Boundary) and temporal distribution of dated samples (OxCal function KDE_Plot) in each region. The crosses represent median dates of these distributions and of individual samples (gray: calibrated individually; black: modelled dates), providing an impression of differences in data density between regions.

**Figure 6:**
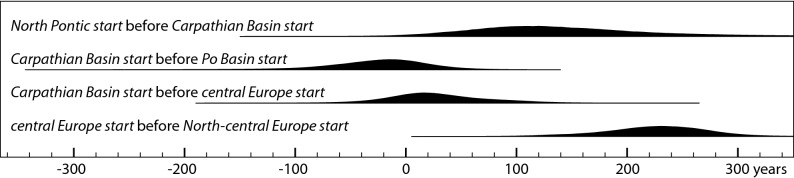
Differences between the estimated start dates of millet cultivation (Fig. 4) in various regions. Positive values indicate that millet cultivation began earlier in the first-named region. The large uncertainty in the *North Pontic start* date (due to low data density in this region) is responsible for the uncertainty in the time lag between the North Pontic and the Carpathian Basin.

The modelled date of introduction of the new crop in each of the sub-regions enables us to chart a relatively clear spatiotemporal sequence of the east–west spread (Figs. 4, 5). Our 'early millet regions' correspond well with the areas associated with distinct material culture or 'cultural groups' and their 'influences', most prominently the Sabatinovka (Caбaтинiвcькa кyльтypa) in the North Pontic area, Tumulus and Trzciniec cultures in eastern, central and western parts of the study region, and the Late Nordic Bronze Age in the north. This re-iterates the previously underlined necessity to tie agricultural/food innovations into material culture and socio-economic change^15,19^, for which a solid temporal frame is instrumental. One of the unexpected discoveries is that, whereas there is almost no measurable difference in the modelled dates of millet appearing in the Carpathian Basin, the Po Basin and central Europe, there was a significant delay (probably > 200 years) before millet spread from central Europe to the north European plain (Figs. 5, 6). A topic for future research is whether this time lag was due to ecological or cultural barriers.

### North Caucasus and North Pontic

The two regions are currently represented by dates from only seven sites, spanning about a millennium. Our preferred model dates the start of millet cultivation in the North Pontic region to the second quarter of the 2nd millennium cal bc (*1650–1500* *cal*bc [68.2% probability] or *1780–1450* *cal*bc [95.4% probability]), and depends on the earliest dates from the Late Bronze Age settlement of Vinogradnyi Sad on the Southern Bug River, occupied c. sixteenth to thirteenth century bc and associated with the Sabatinovka culture, which stretched across the Ukrainian steppe zone^47^ [237, Fig. 79].

Reports of much earlier occurrences (6th–early 3rd millennium bc, Neolithic-Early Bronze Age) of *Panicum miliaceum* in the North Pontic were almost entirely based on millet-like impressions in pottery, daub and figurines^17,48,49^. A recent morphological re-examination of many of these impressions, found on mid–4th or early 3rd millennium bc (Usatovo culture) clay materials from the northwestern Black Sea coast, excluded millet grains as a source for some of the imprints (e.g. those on figurines). Other millet imprints on potsherds and daub were confirmed, along with impressions of wheat and barley grain and few other species^50^. The chronological attribution of this material, however, is insecure and it may in fact date to the 2nd millennium bc^50^ [3209–3210]. Another recent high-resolution assessment of some alleged millet grain impressions in Neolithic, Eneolithic and later pottery from sites in Ukraine has confirmed the origin of the imprints only in the sherds from the Sabatinovka culture sites (1600–1300/1200 bc)^51^.

In the north/northwestern Caucasus, the previously dated large deposit of broomcorn millet grain in Guamsky Grot rock shelter southeast of the Azov Sea confirms the presence of millet here at the very end of the 2nd/beginning of the 1st millennium cal bc^52^. The growing stable isotope evidence from pre- and Early–Middle Bronze and Early Iron Age sites (4th–1st millennium bc) in the region indicates that the local pastoralist groups likely adopted millet into their diet at the end of the 2nd millennium bc (the regional turn from the Late Bronze to the Early Iron Age). This is the time when the high-altitude settlement system was relocated to the valleys and the economic focus shifted towards agriculture^53–55^.

Impressions on the base of a pot in a Kurgan-grave from the second half of the 4th millennium bc (Maykop culture) near Stavropol were described as possibly deriving from millet grains, but closer examination was not carried out^56^ [160]. The dates from Guamsky Grot and the presence of broomcorn millet at some Late Bronze Age sites in the northwest Caucasus (Adygea^57^ [Table 2]) and the Don River delta (Safyanovo^58^ [73]) have been used to suggest that the northward diffusion of the northwest Caucasian Kobyakovo culture (1400/1300–1000 bc) brought the crop into this region. These communities built permanent domestic structures and cultivated crops^52,53^ [256],^58^.

The earliest date on broomcorn millet from Vinogradnyi Sad gives the impression that the crop reached southwestern Ukraine earlier than the northwestern Caucasus. The few early grain-dates from Ukraine cannot clarify this. Potentially earlier dates for the first millet occurrences in the southern Caucasus—late first half, or middle of the 2nd millennium bc^59,60^—leaves a possibility of broomcorn millet appearing at a similar time in the southern and northern Caucasus. The intensive interaction that "crisscrossed the whole of the Caucasus and extended well beyond its boundaries"^61^ [380] would have facilitated this, especially from the mid-2nd millennium bc onwards (Late Bronze–Early Iron Age) when it was promoted by increasing production and circulation of metal objects and metalworking skill^61^ [378–379, 422].

Kobyakovo communities occupied the area along the Kuban and the lower reaches of the Don rivers, neighboured by the Sabatinovka culture area to the west. To the northeast and east, the upper Don and the middle and lower Volga and Ural basins were occupied by the Srubnaya culture (1900–1200 bc)^47,62^. To the east spread the Andronovo cultural complex. The Srubnaya-Andronovo horizon was "a bridge of related cultures [that] extended across the Eurasian steppes from the borders of China and Central Asia to the eastern edge of Europe"^62^ [5]. The cultural borders were porous and the interactions diverse across this vast region used by mobile pastoralists and agriculturalists^62–64^. Perhaps millet took this 'northerly' route from the Inner Asian Mountain Corridor to Ukraine, across northern central Asia^46^ [Fig. 6] and following the latitudinal steppe–forest steppe gradient in northern Kazakhstan (southwestern Siberia). For the latter region, however, archaeobotanical and stable isotope data do not indicate broomcorn millet cultivation and/or consumption before the very end of the 2nd millennium bc or later^65,66^. From current evidence, therefore, millet appears to have arrived in Europe via the "Caucasus corridor", amidst strengthened connections between the two sides of the mountain in the Late Bronze Age^67^ [20, 21]. The early dates of broomcorn millet from southwestern Ukraine are consistent with the several-centuries earlier direct-dates from southern Central Asia (Adji Kui 1 and Ojakly^30,68^) and the Inner Asian Mountain Corridor (Begash^28^).

The stable isotope picture for humans and animals of the 3rd and 2nd millennium bc (Early–Middle Bronze Age) in the North Pontic and the lower Don and Volga region displays isotopic variability attributed to seasonal movements between pastures, diversity of dietary sources, and changing climatic conditions that shaped the local vegetation^69^. Modern steppe vegetation in this area comprises of C_3_, mixed C_3_/C_4_ and C_4_ pastures, whose extent and composition vary depending on the season and climatic characteristics^70,71^. In the second half of the 3rd millennium bc, there seems to have been a period of increased aridity here, which would have favoured C_4_ flora^72^. Elevated δ^13^C values in human and animal bone collagen were explained by the seasonal consumption of C_4_ plants naturally growing in the area,^70–73^. In the Yampil region on the Middle Dniester river (in the steppe zone), several individuals from the Early–Middle Bronze Age graves (late 3rd-first quarter of the 2nd millennium bc) had collagen δ^13^C values of c. − 19.5‰ and were interpreted as possible millet consumers^74^. However, δ^13^C values more negative than − 18‰ do not indicate inclusion of C_4_ plants in the diet^46^.

The now dated presence of millet from the mid-2nd millennium bc onwards in southern Ukraine shows that, from this point on, consumption of this crop could have represented the main source of C_4_ forage and/or food both in coastal areas and, especially, inland. Indeed, in central and eastern Ukraine, a systematic increase in δ^13^C values is seen from the Bronze to the Iron Age, and significantly in the Early Iron Age and the Scythian period^69^ [266, 273],^75,76^. Broomcorn millet grains from several Iron Age sites have been radiocarbon-dated in this and a previous study^77^. Along with the coeval evidence of soil biomarker (miliacin) indicating millet cultivation^13^, they confirm that that the Iron Age isotope data most probably reflect consumption of *P. miliaceum*.

### Carpathian Basin, eastern Alps and Po Basin

Sabatinovka culture communities (1600–1300/1200 bc) in the Pontic steppes and along the Black Sea coast cultivated and consumed millet. At the time, the transect from the upper Dniester to the lower Danube and Transylvania, and further into southeastern Romania and northeastern Bulgaria, was the area of the Noua-Sabatinovka-Coslogeni cultural complex, a tradition seen as an "eastern intrusion"^78^ [889] that maintained trade in metal objects with much of central-eastern Europe^79^ [228–232]. Broomcorn millet grains were recorded at the sites occupied by these groups^80^, which could have served as a medium for the transmission of broomcorn millet further to the west and south. The westward spread could have proceeded up the Danube, through long-established communication and exchange networks via which a number of other goods were distributed, such as metals, amber, and decorative objects^79^ [45–57],^81,82^ [164–196],^83^.

The assumed Danube route may have started already in the late sixteenth/early fifteenth century cal bc, as our preferred model dates the start of millet cultivation in the Carpathian Basin to the early–mid fifteenth century cal bc (*1480–1420 cal*bc [68.2% probability] or *1510–1400 cal*bc [95.4% probability]) (Figs. 4, 5). This was the beginning of the Late Bronze Age (eastern Carpathian Basin) or the end of the Middle Bronze Age/transition to the Late Bronze Age (western Carpathian Basin). In both cases, this is the time of the Tumulus culture, characterized by intensive cross-regional (east–west) connectivity and marked socio-economic change^84^ [125],^85,86^. Here the human stable isotope studies showed a significant increase in δ^13^C values, relative to the previous periods, in the second half of the 2nd millennium bc^87^. Early and Middle Bronze Age archaeobotanical assemblages from the wider region include rare finds of millet^88^, but these have not been dated. In contrast, the assemblages from the second half of the 2nd millennium bc, particularly the tail-end of the Late, and the Final Bronze Age (last quarter of the 2nd millennium bc^85^), frequently contain remains of broomcorn millet^32,39,88,89^ [74,75]. Sometimes these are concentrated grains, such as in one of the ceramic vessels hoarded in a pit at the Tumulus culture site of Lozorno, north of Bratislava in Slovakia^3^.

In the southwestern part of the Carpathian Basin, up the Sava river, the possible early occurrence of broomcorn millet at Mačkovac-Crišnjevi corresponds well with the early dates from the Po Basin to the west. Our model dates the start of millet cultivation in the Po Basin to the early-mid fifteenth century cal bc (*1500–1430 cal*bc [68.2% probability] or *1570–1410* cal bc [95.4% probability]), which coincides with the start of the fully developed Terramare culture (1550–1150 bc^90^). The stable isotopic ratios of humans point to the mid-2nd millennium bc for the start of millet consumption in northern Italy; by the mid-fifteenth century bc, the Terramare communities consumed considerable amounts of millet^44,91,92^. The grains and chaff (glumes) of broomcorn millet are frequent and abundant in the archaeobotanical assemblages from this period onwards^93,94^ [42,114,115]. The transfer of goods (e.g. swords, amber) between the Carpathian Basin and the Po Basin/northern Italy (through lowland Austria and Slovenia) during the Bronze Age has long been recognized^79^ [77],^84^ [127],^95,96^. It seems that the Carpathian Basin acted as a broomcorn millet 'hub' from where the crop radiated in different directions, including south across the central/western Balkans and into northern Greece^40,97^.

The increased socio-political and economic connectivity across the Mediterranean in the second half of the 2nd millennium bc^81,82,84,96,98^ perhaps facilitated a 'southerly' route of transfer of millet into peninsular Italy and the Balkans, with the city of Mycenae as the potential "power node"^94^ [87]. However, the majority of Late Bronze Age (1600–1100 bc) archaeobotanical finds of broomcorn millet in Greece are located in the north, including several large deposits^40^, of which the one at Assiros was direct-dated to the fourteenth century cal bc (1405–1305 cal bc [95.4% confidence], refined to *1374–1339 cal*bc [95.4% probability] according to the published site chronological model)^99^. In contrast, only one broomcorn millet find has so far been reported in the south, consisting of ten (undated) grains from the Mycenaean palace of Tiryns^100^. A C_4_ signal was detected in the stable isotopic composition of human and animal bones from mid-2nd millennium bc Greece, with millet and other C_4_ plants stated as possible contributors^101–103^. The presence of broomcorn millet grains in the mid-late 2nd millennium bc layers of Troy^104^ [9] perhaps point to Anatolia as a source area for the introduction of the crop in the Aegean. On the other hand, the archaeological evidence (e.g. amber and metal objects) documents earlier interactions between the Tumulus culture societies of the Carpathian Basin and the Aegean city-states^84,105^ [361]. Further radiocarbon dates on broomcorn millet from the southeast corner of Europe will elucidate its diffusion into this region.

### Central Europe (north of the Carpathians and the Alps)

According to our preferred model, broomcorn millet was probably first cultivated in central Europe in the mid-fifteenth century cal bc (*1470–1400 cal*bc [68.2% probability] or *1490–1330 cal*bc [95.4% probability]), soon after its appearance in the Carpathian Basin. It seems to have appeared almost simultaneously in southern Poland, the Czech Republic and southern Germany. A 'northerly' route of its arrival into southern Poland is possible, through the forest steppe of northern Ukraine, up the rivers flowing into the Black Sea. For much of the 2nd millennium bc, the middle and upper courses of the Prut, Dniester, Southern Bug and Dnieper were characterized by the Middle–Late Bronze Age Trzciniec culture (1800–1100 bc^106–108^). The fourteenth/thirteenth century bc Trzciniec sites east of the northern Carpathian foothills show similarities in pottery and metal objects with the Noua and Sabatinovka cultures. These intercultural contacts^107,109^ [272–278] may have incorporated crop exchange. The regions north of the Alps (the upper Danube and the upper Rhine, including southern Germany, Bohemia and parts of Austria) were identified as the area from which the Tumulus culture spread eastwards to the Tisza and the upper-middle Oder and Vistula rivers^82^ [97],^84^ [125],^106,108^. Perhaps rather than arriving from the 'east' into southern Poland, broomcorn millet moved west-to-east, along the northern Carpathian foothills and onto the loess plateaus, such as the one on which the millet-rich site of Lipnik was located (1400–1100 cal bc).

There are striking similarities in the mid-2nd millennium bc pottery shapes and styles in the "communication corridor" running along the Tisza river, crossing the Carpathians and connecting their southern basin and northern slopes^108^. We dated broomcorn millet from the site of Maszkowice in the western part of the northern Carpathian foothills, where the characteristics of pottery and metal finds correspond to those seen in the south of the Tisza valley, within the Otomani-Füzesabony cultural tradition of the Carpathian Basin^108^. These contacts hint at the potential south-north direction of the crop dispersal. The Vistula river tributaries flowing down the northern Carpathian foothills, and the Morava and Oder river valleys were recognized as the communication channels through which goods and/or people moved between the Carpathian Basin and southern Poland, and further to the north^107^ [199]. We may be seeing several different, possibly synchronous, dispersal routes of millet within and beyond the matrix of individual cultural entities.

Our estimated start date for millet cultivation in central Europe is consistent with archaeobotanical data from sites in Bohemia, which place the first occurrence of broomcorn millet here at the start of the regional Middle Bronze Age (1600–1250 bc^110^ [267],^111^). Stable isotope results from animals and humans from sites in Bohemia and Moravia suggest a striking change in diet between the early and late 2nd millennium bc, with Late Bronze Age diets having a significant contribution of millet^112^. The age of the recently reported miliacin in soil from a fissured ceramic vessel found in a Corded Ware culture (3rd millennium bc) grave at Držovice, central Moravia^113^ is highly questionable. The soil in the pot contained traces of miliacin, but the soil around the pot was not tested and so the possibility of contamination cannot be excluded, particularly since the grave was damaged by modern digging of a drainage system^113^ [4222],^114^ [188]. Without an absolute date for the miliacin itself, it is possible that the association is coincidental and that the miliacin is more recent than the pot. There are as yet no direct dates on broomcorn millet from sites in Moravia but, assuming the east–west transfer through the Danube Basin, it could have reached southern Moravia some time before it is found in Bohemia, the Alpine Foreland and the upper reaches of the Rhine.

The broomcorn millet grain from the Corded Ware site of Binningen, located in the northern foothills of the Jura mountains north of the Western Alps, gave a twelfth century cal bc date (Supplementary Dataset). The isotopic investigations of animals and humans from three sites in southwestern Switzerland revealed ^13^C enrichment at the end of the 2nd or start of the 1st millennium bc (Final Bronze Age, 1300–800 bc^115^). Earlier dates (fifteenth/fourteenth century cal bc) on broomcorn millet from Lauda-Königshofen in the upper Danube basin and Bruchenbrücken on the upper-middle Rhine show that millet had already been cultivated in adjacent regions for some time. Lauda-Königshofen was the location of a Corded Ware culture cemetery, where the remains of later (metal-age) settlements were also found. The broomcorn millet grains originate from the Corded Ware layers, but are obviously intrusive. Stable isotope results from humans buried here in the mid-3rd millennium BC gave no indication of consumption of C_4_ plants^116^.

### North-central Europe (northern Germany and northern Poland)

There are now forty radiocarbon dates on broomcorn millet from twenty-five sites in northern parts of Germany, and this is the best-covered region so far. Additional sites in north-central Poland add to the regional picture. The beginning of broomcorn millet cultivation in this region is dated to *1230–1160 cal*bc (68.2% probability) or *1260–1140 cal*bc (95.4% probability). Considering the number of relatively early sites and samples dated (Supplementary Dataset), it is unlikely that dating more sites will produce significantly earlier dates. By the mid-twelfth century bc, broomcorn millet was present in the entire region, suggesting a rapid spread over the lowlands (Figs. 4, 5, 6). The coupled biomolecular and isotopic analyses on food remains from a Late Bronze Age (1100–800 cal bc) horizon at the site of Bruszczewo in northern Poland testify to its consumption^117^.

The thirteenth century bc was the time of appearance of the Urnfield culture in central Europe, which, in the twelfth century bc spread to the south and the north^79^ [31],^82^ [Fig. 1.2],^118^. Within this general phenomenon, many regional cultural groups have been differentiated, such as the Lausitz/Lusatian culture in central Europe whose influence spread to the north^79^ [346],^119^. Northern European communities maintained communication with the Carpathian Basin and Tumulus culture groups throughout the 2nd millennium bc, particularly during its first half^120^. In the second half of the millennium, the interaction extended beyond (and via) the Tumulus culture zone, to the Aegean city-states and was likely centered on amber commerce, along with trade in metals and other goods^96,105^ [365],^120^ [614]. Agricultural products could have been transmitted through any or all of these trans-regional networks. It has been suggested that broomcorn millet arrived into North-central Europe through the trade routes following major rivers – the Oder, Elbe and Weser^121^. Smaller waterways were probably also part of this network, such as the Tollense in northeastern Germany, where palaeodietary isotopic evidence (seldom available from the region and period due to the prevalent cremation ritual) shows that victims of a Late Bronze Age battle were millet consumers^122,123^ (Supplementary Text 2, Supplementary Fig. 5].

### Millet beyond *When* and *Where*

Archaeological scholarship on the origin of domesticated broomcorn millet and its journey along the c. 8,000 km east–west Eurasian route(s), as well as the environmental and social circumstances at different stages of these process, has grown at an incredible pace, with the major focus on the evidence from Asia. In Europe, research on millet has not taken place at a similar scale, although some authors suggested possible routes and mechanisms of its introduction^40^. In the wider context of the long-lasting 'trans-Eurasian exchange^20^ of many different elements, including raw materials, technologies, objects, animals and diseases^64,124^, broomcorn millet documents the process of 'food globalisation' in the Old World^17,21–23,26,125^. Reconstructing the *When* and *Where* of broomcorn millet in Europe helps us to understand whether the crop arrived “with a bang”^126^ or if it ‘trickled in’ over the millennia of archaeologically well-documented communication between the east and the west. Europe in the Middle and Late Bronze Age is a paragon of connectivity and intercultural influences, which were particularly intensive in the second half of the 2nd millennium bc. Without doubt, the amplified cross-regional interaction for the purpose of trade/exchange served as a catalyst for the transmission of agricultural innovations, including broomcorn millet, which perhaps was one of the traded goods.

Perhaps the knowledge of millet's agro-ecological requirements and related culinary traditions travelled along with it and contributed to its relatively swift transmission between distant regions. Thanks to its versatility and ecological and functional ‘plasticity’, broomcorn millet may have become highly valued asset, especially in changing or challenging environmental and social settings. There are, however, indications of ‘non-adoption’ or, more precisely, non-consumption of millet among some communities at the time when their neighbours consumed notable amounts of it. This was seen in the human stable isotopes from the Bronze and Iron Age Trans-Urals^66^, the Terramare culture zone^91^ and Iron Age coastal Croatia^116^. Thus, at a more local scale, the introduction and use of the new crop may not have been indiscriminate^43^. Various cultural reasons may have been at play, determining the (degree of) acceptance or rejection of broomcorn millet. To explore this further, it is important to understand the economic, technological and social environments into which broomcorn millet arrived in Europe, alongside the ones it may have created.

## Conclusions

This study produced and evaluated over 100 radiocarbon dates directly on charred broomcorn millet grains from over 90 sites in Europe. The main aim was to test a substantial number of grains recovered from a range of archaeological deposits in Europe attributed to the Neolithic, Copper and Bronze Ages and, in particular, high-density millet deposits (grain concentrations) attributed to the Bronze Age. This dataset decisively pinpoints the middle of the 2nd millennium bc (Middle/Late Bronze Age) as the time of the earliest occurrence of broomcorn millet in the studied regions of Europe. The crop emerged at only slightly different times in different parts of Europe, earliest in Ukraine, as expected given its eastern origin. Within about a century of its appearance in the Carpathian Basin and northern Italy, broomcorn millet was found throughout central Europe. It was not immediately diffused into lowland northern Germany, however, and it is only in the late thirteenth/early twelfth century cal bc that it spread rapidly through this region.

The radiocarbon dates show that the new crop spread fast and was found in large quantities immediately or soon after its introduction in different parts of Europe. The abundance of millet has been taken as a reflection of its cultivation. The period for which the stable isotope evidence first hints at the consumption of broomcorn millet overlaps with the earliest radiocarbon-dates on both small and large archaeobotanical records of the crop. Whereas inter-and intra-regional variations in this pattern can be expected, a general impression is one of a relatively swift and potentially comprehensive adoption of the new crop at the continental level.

Broomcorn millet transfer epitomises the process of 'food globalisation' in the Old World. Not just the routes and pace of this process have been of interest. A host of additional questions have been brought forward concerning the mechanisms, cultural and environmental context of the spread, as well as the impact of the new crop on agrarian production, animal husbandry and food consumption in the source, transit and receiving regions. Sweeping changes in economy, social relations and ideology have been archaeologically documented in mid-late 2nd millennium bc Europe. Against this backdrop, can we now talk about a 'millet effect'? Some of the characteristics of this crop, principally its short growth-cycle and surplus potential, would have had wide ramifications in aspects such as intensity and scale of agricultural production, labour organisation, land tenure, food storage, culinary practices and, ultimately, social and political power. With the time of the arrival of the crop to the western end of the “Eurasian dyad” now determined, we can address the transformative power of millet from a solid chronological ground.

## Materials

Published archaeobotanical and radiocarbon studies^17,18,27,39,40,48,127^ provided an overview of the material potentially available for dating. The study period and regions—Neolithic and Bronze Age periods in eastern, central and North-central Europe—were defined in line with the aims and scope of the Collaborative Research Centre SFB 1266: *Scales of Transformation* at Kiel University, the time and funding available through this initiative in 2017–2019, and the working assumptions that broomcorn millet arrived in the Neolithic and that its full-fledged cultivation started in the Middle/Late Bronze Age. The dating programme had two objectives: (1) radiocarbon dating of ‘early’ finds of broomcorn millet reported from Neolithic and Copper Age sites and typically consisting of one-few grains, in order to date the first introduction of the crop in different parts of the study region; (2) radiocarbon dating of Bronze Age finds of millet, specifically grain concentrations, in order to temporally correlate this agricultural innovation with developments in lifestyle, economy and social relations characteristic of the period.

Based on their research focus and publications, archaeobotanists working in the parts of Europe targeted by the programme were informed about the initiative. They were sent guidelines for the selection and submission of the specimens for dating, along with a questionnaire (Supplementary Sample form). The material was received in Kiel, where the specimens for dating were selected and photographed (see examples in Fig. 2) and the relevant information entered in the 'Millet Dating Programme' data repository.

## Methods

### Sampling and AMS-dating

We dated charred grains from 'small' and 'large' broomcorn millet deposits (Supplementary Dataset) from relatively or absolutely dated Neolithic through Bronze Age layers. We did not intentionally sample grains from contexts/sites dated to later periods. Small deposits comprise one to several dozen grains, usually dispersed throughout an excavation unit/flotation sample. Many of these contexts (feature/layer) were stratigraphically dated to the late 7th to mid-3rd millennium bc (broadly the Neolithic and Copper Age in Europe). Large deposits comprise tens to thousands of grains from a discrete context (e.g. burnt layer or lens, pit/oven/hearth fill, pot content), often high-density concentrations of macro-plant remains predominantly or entirely composed of broomcorn millet, sometimes agglutinated (fused together) during charring.

Where more than one grain was available, we selected the two largest and best-preserved grains for dating (the second as a reserve). We normally dated single grains, but in several cases we dated two or more agglutinated grains, because these grains cannot have been significantly different in date, since they were uncharred before they were fused together. This study also includes a few ^14^C dates on bulk samples of two to several millet grains from small deposits, homogenized by pulverisation (see Supplementary Dataset). If these samples consisted of grains of different dates, the calibrated ^14^C result must be more recent than the earliest millet grain present; such results are therefore still informative in terms of dating the first appearance of millet. We dated samples at the Accelerator Mass Spectrometry (AMS) laboratories in Poznań (Poznań Radiocarbon Laboratory, Poland) and Kiel (Leibniz Laboratory for Radiometric Dating and Stable Isotope Research, Germany), following standard laboratory protocols for charred plant macrofossils (Poznań^128^ [ZR]; Kiel^129^). This facilitated the incorporation of previously obtained (un)published dates on charred broomcorn millet grains (Supplementary Text 1).

### Chronological modelling

We used Bayesian chronological modelling to visualize spatial patterns in the earliest dates^130^. This approach is based on the premise that while the dates of our samples are constrained by when millet cultivation began in any region, it is highly unlikely that our samples include the first grains of broomcorn millet grown at any site, or even that they come from the earliest millet-growing site in each region (Supplementary Text 2; Supplementary Fig. 2). We used OxCal v.4.3^35^ and the IntCal13 calibration curve^131^ to convert ^14^C ages to calendar dates, and the OxCal Bayesian chronological modelling functions. The model’s posterior density estimates of the dates of samples and events, and of intervals between these dates, are reported *in italics*, to distinguish them from independently calibrated individual dates. The OxCal CQL model code is provided as Supplementary Model code, allowing readers to reproduce our models and improve them with new data (the OxCal software required to run this model is freely available^132^).

## Supplementary information

Supplementary Dataset.

Supplementary Figure 1.

Supplementary Figure 2.

Supplementary Figure 3.

Supplementary Figure 4.

Supplementary Figure 5.

Supplementary Information 1.

Supplementary Information 2.

Supplementary Information 3.
