# Supplementary material for: New AMS 14C dates track the arrival and spread of broomcorn millet cultivation and agricultural change in prehistoric Europe
Source: Sci Rep. 2020 Aug 13;10:13698. doi: 10.1038/s41598-020-70495-z (PMC7426858; doi:10.1038/s41598-020-70495-z)
Supplement: Supplementary file 7 — Supplementary Information 1. [file 41598_2020_70495_MOESM7_ESM.docx]

Supporting Information for

**New AMS ^14^C dates track the arrival and spread of broomcorn millet cultivation and agricultural change in prehistoric Europe**

Dragana Filipović, John Meadows, Marta Dal Corso, Wiebke Kirleis, Almuth Alsleben, Örni Akeret, Felix Bittmann, Giovanna Bosi, Beatrice Ciută, Dagmar Dreslerová, Henrike Effenberger, Ferenc Gyulai, Andreas G. Heiss, Monika Hellmund, Susanne Jahns, **Thorsten Jakobitsch,** Magda Kapcia, Stefanie Klooß, Marianne Kohler-Schneider, Helmut Kroll, **Przemysław Makarowicz,** Elena Marinova, Tanja Märkle, Aleksandar Medović, Anna Maria Mercuri, Aldona Mueller-Bieniek, Renato Nisbet, Galina Pashkevich, Renata Perego, Petr Pokorný, Łukasz Pospieszny, Marcin Przybyła, Kelly Reed, Joanna Rennwanz, Hans-Peter Stika, Astrid Stobbe, Tjaša Tolar, Krystyna Wasylikowa, Julian Wiethold, Tanja Zerl

Corresponding author: Dragana Filipović

Email: d.filipovic@ufg.uni-kiel.de

Corresponding author: John Meadows (Bayesian chronological modelling)

Email: jmeadows@leibniz.uni-kiel.de

The Supporting Information consists of:

**Supplementary Text 1**. Radiocarbon Dating. Bayesian chronological modelling.

**Supplementary Text 2**. Sensitivity testing. The date of the Tollense battle.

**Supplementary Figure 1.** Relationship between graphite weight and AMS δ^13^C values in samples measured for this paper by the Poznań Radiocarbon Laboratory. δ^13^C values are consistent with target-size-dependent fractionation during AMS measurement of broomcorn millet samples.

**Supplementary Figure 2**. Schematic structure of our Bayesian chronological model. Top: assumed temporal distribution of samples from each region (falling exponential distribution). Below: principal OxCal CQL functions^5^, shown in equal-area font. Each region is treated independently.

**Supplementary Figure 3**. Kernel-density estimates^8^ of the temporal distribution of (a) all directly-dated millet grains predating 1 cal BC; (b) dated grains from small deposits only; (c) dated grains from large deposits only. Note that (b) and (c) are subsets of the modelled dates included in (a), not separate models.

**Supplementary Figure 4**. Sensitivity testing: (left) variation in median estimated start date for each region, according to assumed temporal distribution of all pre-Roman dates from that region (Tau_Boundary [preferred model], falling exponential distribution; Zero_Boundary, falling ramped distribution; Sigma_Boundary, falling sigmoid distribution; Boundary, uniform distribution)^5^; (right) variation in median estimated start date for each region, depending on exclusion of some of the most recent samples (we omitted dates after 2000 BP from the preferred model).

**Supplementary Figure 5**. Bayesian chronological model in OxCal v.4.3^5^ for the date of the Tollense battle. Outline distributions are simple calibrations of radiocarbon ages of samples apparently associated with the 'conflict horizon'^9,13–15^. Black distributions are posterior density estimates of sample formation date. To allow for minor wood-age offsets and collagen turnover time, the model requires these samples to have formed over a period of 20 ± 10 calendar years (using the OxCal Span function), which ended with the date of the battle (estimated using the Last function; red). Gray distributions are simple calibrations of samples omitted as outliers (including these results would not affect the estimated battle date). The dendrochronological dates of *in-situ* structural timbers from Tollense site Weltzin 13, shown below (green), are not included in this model. The estimated battle date can also be compared to the start of millet cultivation in this region (pink; see Fig. 5). Simulation exercises show that including up to 22 unpublished ^14^C ages for arrow shafts (only simple calibrations of these results are published^15[Abb.15)^) would not significantly affect our battle date estimate. Dietary reservoir effects appear to have been negligible, but any correction applied would produce a later battle date.

**Supplementary Dataset**. Details on the broomcorn millet samples and their ^14^C dates produced or considered within this study, along with some basic information on the archaeological contexts and sites from which the material derived. The readers should consult this in conjunction with figures and tables in the text and supporting materials.

**Supplementary References**. Literature cited in: Supplementary Text 1, Supplementary Text 2, Supplementary Figures 2-5 and Supplementary Dataset.

**Supplementary Sample form**. Sample information form completed for each sample dated through the CRC1266 Millet Dating Programme.

**Supplementary Model code**. Users should copy the OxCal v 4.3 Chronological Query Language code and paste it into the input window, save it as an OxCal project file (.oxcal) and run it. OxCal is freely available at https://c14.arch.ox.ac.uk/oxcal/OxCal.html.

This file includes Supplementary Text 1, Supplementary Text 2 and list of Supplementary References

Supplementary Text 1

**Radiocarbon dating**

Samples were extracted following standard laboratory protocols for charred plant macrofossils (Poznan^1[ZR]^; Kiel^2^). Charred grains were first acidified (with 1% HCl) to remove secondary carbonates, then treated in a hot alkali solution (1% NaOH at 60 °C) to dissolve mobile organic compounds, and then acidified again (with 1% HCl). The insoluble residue (humin) was dated. Our Bayesian model also incorporates the dates obtained by^3^, who applied a less aggressive pre-treatment protocol (skipping the alkali and re-acidification steps) in order to maximize the extracted carbon yield.

The extract was combusted in an evacuated quartz tube with CuO and silver wool at 900 °C. The resulting CO_2_ was reduced to graphite for measurement by AMS. Kiel used a 3 MV HVEE Tandetron AMS, in operation since 1995 and upgraded in 2015. At Poznań, samples were dated either on an NEC 1.5 MV Pelletron AMS used since 2001, or a second compact NEC system installed in 2013. AMS systems measure ^12^C, ^13^C and ^14^C ion currents from each graphite target simultaneously. The ^13^C/^12^C ratio (AMS δ^13^C) is used to normalize the ^14^C current for natural and instrumental fractionation, and thus to calculate conventional ^14^C ages (Supplementary Fig. 1). The reported ^14^C age errors incorporate uncertainties in measurement, standard normalization, instrumental background, blank correction, and additional uncertainty arising from sample pretreatment, based on long-term experience with laboratory standard and known-age samples of similar materials. Supplementary Figure 1 shows that, although the AMS δ^13^C values for many samples are relatively low for C_4_ plants, this pattern is simply a function of sample size. It does not affect the ^14^C ages, because the AMS δ^13^C values are used to correct ^14^C currents for fractionation as part of the normal ^14^C age calculation.

**Bayesian chronological modelling**

Bayesian chronological modelling uses complex algorithms to combine ‘standardized likelihoods’ (here the calibrated ^14^C ages) with ‘prior information’ about the relative dates of samples and associated events. Although we have no archaeological evidence that any one sample was the same date, earlier or later than any other, we know that their dates are not entirely independent of each other, as all the dates are on millet, whose dispersal in prehistoric Europe is regarded as a unique phenomenon, whose beginning and tempo may have varied regionally. This means that *a priori*, we expect the dates from each region to be clustered in time, with an overall temporal distribution determined by when millet cultivation began, and how successful our program was at obtaining samples dating close to the start of millet cultivation. By applying an expected overall temporal distribution, Bayesian chronological modelling allows the scatter of millet dates from multiple sites to be used to estimate when millet cultivation began in each region.

Our preferred model (Supplementary Model code) is formally similar to that used to map the spread of wheat in Asia^4^, as we assumed that, given our sampling strategy, ‘early’ samples would date close to the start of millet cultivation, whereas there would be a long tail of (much) later dates expected from Bronze Age contexts and also due to potential intrusion of later grains. We implemented this assumption by applying a falling exponential distribution to the dates included in the model (Supplementary Fig. 2) (OxCal function Tau_Boundary^5^). By contrast, another study^6^ used the more traditional ‘uniform span prior’ model (OxCal function Boundary^7^) to interpret early millet dates in northeast Asia, applying a uniform distribution to sample dates (i.e. assuming that earlier and later samples are equally probable). In common with both case studies, we did not build individual site chronologies, as nearly all sites are represented by only 1 or 2 dates; instead, we regarded each sample as randomly drawn from a falling exponential temporal distribution. To test which model was more appropriate to our data, we used kernel-density estimation (Supplementary Fig. 3) to summarize the temporal distribution of sample dates independently of these assumptions^8^. We also compared the output of our preferred model to that of models based on uniform and other possible distributions of sample dates (sensitivity testing).

A second aspect of the model subjected to sensitivity testing was the attribution of sites to regions. The spatial distribution of dated samples is biased by research history and does not represent the full geographic extent of millet cultivation, any more than it covers the exact period of millet cultivation. Regional groupings of dated sites are therefore essential for spatial-temporal modelling to be meaningful. Ideally, we would compare the start of millet cultivation in many small equally sized regions (cells), but dated sites can be tightly clustered or widely spread, and cells would not include any early sites, so there is inevitably a trade-off between spatial resolution and temporal precision. On the other hand, we are not dealing with an obviously discontinuous spatial distribution of dated sites which imposes its own regional clusters. Thus, any regional subdivision of sites involves arbitrary compromises. Our preferred regional subdivision (Fig. 3) – which broadly reflects archaeological cultural groupings – was reached after comparing model output from various permutations of the data set (with more, smaller regions), and finding that they did not provide additional insights.

Finally, we checked the effect of arbitrarily excluding the most recent dates. We did not set out to date the end of millet cultivation, and most of the later dates obtained are from small deposits of millet dated archaeologically to the Neolithic. These dates appear to confirm that millet cultivation continued through the Roman period into the Middle Ages, but in principle the phenomenon might have been discontinuous. As we need to apply a temporal distribution to the sample dates to estimate the start of millet cultivation, we chose to truncate the data set, in case uncertainty about the end of the phenomenon influenced the estimated start dates. In the preferred model, we only omitted 17 samples dating after c. 1 cal BC, but we also ran the model using all dated samples, and omitting 53 samples with ^14^C ages below 2600 BP (c. 800 cal BC).

Supplementary Text 2

**Sensitivity testing**

Supplementary Figure 4 shows how sensitive the median start date in each region is to different aspects of the chronological modelling process. The start of millet cultivation in each region is regarded as an event (OxCal function Boundary), before which there cannot be any direct dates on millet, but the assumed temporal distribution of samples can be varied, which will slightly affect the estimated start date. We therefore re-ran the model using all temporal distributions available^1^. Our preferred model, with a falling exponential distribution of sample dates, consistently gives the latest median start date in each region, while the traditional uniform distribution produces the earliest median start dates (Supplementary Fig. 4-left). These differences span only a few decades – 50-60 years at most, i.e. within estimated uncertainties – and do not suggest that the apparent direction of diffusion depends on which model is used. Unsurprisingly, the most consistent results are for the Po Basin, where there are no later sites in our dataset. Our preferred model gives the most precise estimates for the start dates, and the uniform distribution gives the least precise estimates. The arbitrary omission of some of the most recent samples appears to have even less impact on estimated start dates than the choice of temporal distribution (Supplementary Fig. 4-right).

**The date of the Tollense battle**

Human remains from the bed of the Tollense have stable isotope values indicative of millet consumption^9–12^. Strontium (Sr) isotopic analysis identified the presence of the remains of local and non-local individuals at the site, with non-locals potentially originating from regions to the south, e.g. Bohemia^12^. Enamel δ^13^C values in both groups suggest millet consumption, but the non-local Sr values are associated with significantly higher millet intake. All individuals with elevated δ^13^C values are regarded as victims of a battle, whose date has been informally estimated as 1200 ± 40 cal BC^9^, c.1250 cal BC^12^, or 1300–1250 cal BC^13^. The 1300–1250 cal BC range, which is quoted in most recent publications^e.g.14^, would appear to slightly predate the start of broomcorn millet cultivation in this region, but Bayesian chronological modelling of the published ^14^C results (Supplementary Fig. 5) suggests a battle date of *1210 ± 10 cal BC*, shortly after the construction of a dendrochronologically dated timber structure on the east bank (c. 1225 ± 6 BC; Supplementary Fig. 5), and in agreement with the modelled date of the start of millet cultivation in north-central Europe.

Supplementary References

Literature cited in Supplementary Text 1, Supplementary Text 2, Supplementary Figures 2-5 and Supplementary Dataset.

1 Brock, F., Higham, T., Ditchfield, P., Bronk Ramsey, C. Current pretreatment methods for AMS radiocarbon dating at the Oxford Radiocarbon Accelerator Unit (ORAU). *Radiocarbon* **52**, 103–112 (2010).

2. Grootes, P.M., Nadeau, M-J., Rieck, A. 14C-AMS at the Leibniz-Labor: radiometric dating and isotope research. *Nuclear Instruments and Methods in Physics Research Section B: Beam Interactions with Materials and Atoms* **223**, 55–61 (2004).

3. Motuzaite-Matuzeviciute, G., Staff, R.A., Hunt, H.V., Liu, X., Jones, M.K. The early chronology of broomcorn millet (*Panicum miliaceum*) in Europe. *Antiquity* **87**, 1073–1085 (2013).

4. Liu, X., *et al.* The virtues of small grain size: Potential pathways to a distinguishing feature of Asian wheats. *Quaternary International* **426**, 107–119 (2016).

5. Bronk Ramsey, C. Bayesian analysis of radiocarbon dates. *Radiocarbon* **51**, 337–360 (2009).

6. Leipe, C., Long, T., Sergusheva, E.A., Wagner, M., Tarasov, P.E. Discontinuous spread of millet agriculture in eastern Asia and prehistoric population dynamics. *Science Advances* **5**, eaax6225 (2019).

7. Bronk Ramsey, C. Comment on 'The use of Bayesian statistics for 14C dates of chronologically ordered samples: A critical analysis'. *Radiocarbon* **42**, 199–202 (2000).

8. Bronk Ramsey, C. Methods for summarizing radiocarbon datasets. *Radiocarbon* **59**, 1809-1833 (2017).

9. Jantzen, D., *et al.* A Bronze Age battlefield? Weapons and trauma in the Tollense Valley, north-eastern Germany. *Antiquity* **85**, 417–433 (2011).

10. Terberger, T., Heinemeier, J. Die Ernährungsweise der bronzezeitlichen Menschen aus dem Tollensetal im Spiegel ihrer 13C- und 15N-Isotopie –Erste Ergebnisse in *Tod im Tollensetal – Forschungen zu den Hinterlassenschaften eines bronzezeitlichen Gewaltkonfliktes in Mecklenburg-Vorpommern* (eds. Jantzen, D., Orschiedt, J., Piek, J., Terberger, T.) 209–214 (Landesarchäologie MV, 2014).

11. Price, T.D. Isotopic analysis of human tooth enamel from the Tollense Valley, Germany. A preliminary report in *Tod im Tollensetal – Forschungen zu den Hinterlassenschaften eines bronzezeitlichen Gewaltkonfliktes in Mecklenburg-Vorpommern* (eds. Jantzen, D., Orschiedt, J., Piek, J., Terberger, T.) 223–232 (Landesarchäologie MV, 2014).

12. Price, T.D., *et al.* Multi-isotope proveniencing of human remains from a Bronze Age battlefield in the Tollense Valley in northeast Germany. *Archaeological and Anthropological Sciences* **11**, 33–49 (2019).

13. Jantzen, D., *et al.* An early Bronze Age causeway in the Tollense Valley, Mecklenburg-Western Pomerania–The starting point of a violent conflict 3300 years ago? *Bericht der Römisch-Germanischen Kommission* **95**, 13-49 (2017).

14. Uhlig, T., *et al.* Lost in combat? A scrap metal find from the Bronze Age battlefield site at Tollense. *Antiquity* **93**, 1211–1230 (2019).

15. Terberger, T., *et al.* Das bronzezeitliche Kampfgeschehen im Tollensetal–ein Großereignis oder wiederholte Konflikte in *Bronzezeitliche Burgen zwischen Taunus und Karpaten Universitätsforschungen zur prähistorischen Archäologie* (eds. Hansen, S., Krause, R.) 103–124 (Habelt, 2018).

16. Wardle, K., Higham, T., Kromer, B. Dating the End of the Greek Bronze Age: A Robust Radiocarbon-Based Chronology from Assiros Toumba. *PLOS One* **9**, e106672 (2014).

17. Trifonov, V.A., Shishlina, N.I., Lebedeva, E.Yu., van der Plicht, J., Rishko, S.A. Directly dated broomcorn millet from northwestern Caucasus: Tracing the Late Bronze Age route into the Russian steppe. *Journal of Archaeological Science: Reports* **12**, 288–294 (2017).

18. Kirleis, W. *Atlas of Neolithic plant remains from northern central Europe* (Barkhuis Publishing, 2019).

19. Hellmund, M. Frühneolitische Anbau von Rispenhirse in Mitteldeutschland? – Der eisenzeitliche Rispenhirsefund von Hundisburg, Lkr. Börde in *Archäologie in Sachsen-Anhalt 6* (eds. Meller H., Weber, T.) 297–303 (Landesamt f. Denkmalpflege u. Archäologie Sachsen-Anhalt, 2012).

20. Kapcia, M., Mueller-Bieniek, A. An insight into Bronze Age subsistence strategy in forested Carpathian foothills, based on plant macro-remains. *Archaeological and Anthropological Sciences* **11**, 2879–2895 (2019).

21. Mueller-Bieniek, A., Kittel, P., Muzolf, B., Muzolf. P. Useful plants from the site of Lutomiersk-Koziówki near Łódź (central Poland) with special reference to the earliest find of *Xanthium strumarium* L. seeds in Europe. *Journal of Archaeological Science: Reports* **3**, 275–284 (2015).

22. Kirleis, W. Vegetationsgeschichtliche und archäobotanische Untersuchungen zur Landwirtschaft und Umwelt im Bereich der prähistorischen Siedlungen bei Rullstorf, Ldkr. Lüneburg. *Probleme der Küstenforschung im südlichen Nordseegebiet* **28**, 65–132 (2003).

23. Kirleis, W. Ein goldgelber Schatz: Der älteste Rispenhirsevorrat Niedersachsens. *Archäologie in Niedersachsen* **7**, 42–44 (2004).

24. Harding, A., Šumberová, R., Knüsel, C., Outram, A. *Velim. Violence and Death in Bronze Age Bohemia* (Institute of Archaeology of the Academy of Sciences of the Czech Republic, 2007).

25. Pokorný, P., *et al.* Mid-Holocene bottleneck for central European dry grasslands: Did steppe survive the forest optimum in northern Bohemia, Czech Republic. *The Holocene* **25**, 716–726 (2015).

26. Saalow, L., Schmidt, J.-P., Selent, A. 340 km Ausgrabung – Planung, Durchführung und Ergebnisse der Untersuchung im Trassenverlauf in *Pipeline: Archäologie. Ausgrabungen auf den großen Ferngastrassen in Mecklenburg-Vorpommern* (eds. Dworzak, A., Meier, U.M., Saalow, L., Gräfin von Schmettow, H., Schmidt, J.-P., Selent, A.) 23–38 (Mecklenburg-Vorpommern/Landesamt für Kultur und Denkmalpflege, 2014).

27. Jahns, S., *et al.* Zur Geschichte der nacheiszeitlichen Umwelt und der Kulturpflanzen im Land Brandenburg in *Beiträge zur Archäozoologie und Prähistorischen Anthropologie Band XI (*eds. Flohr, S., Morgenstern, P.) 9–35 (Gesellschaft für Archäozoologie und Prähistorischen Anthropologie e.V., 2018).

28. Schmütz, K. *Die Entwicklung zweier Konzepte. Großsteingräber und Grabenwerke bei Haldensleben-Hundisburg* (Rudolf Habelt, 2017).

29. Regnell, M. Botaniska analyser av jordprover från gravhög i Mörbylånga s:n, Öland in *Storhögen på kustslätten – undersökningen av Risinge hög på Öland 2011* (eds. Papmehl-Dufay, L., Goldhahn, J.) 64–70 (Kalmar Studies in Archaeology, 2018).

30. Гак, Е.И., Антипина, Е.Е., Лебедева, Е.Ю., Кайзер, Э. Хозяйственная модель поселения среднедонской катакомбной культуры Рыкань-3. Р*оссийская археология* ***2***, 19–34 (2019).
