# Supplementary material for: New AMS 14C dates track the arrival and spread of broomcorn millet cultivation and agricultural change in prehistoric Europe
Source: Sci Rep. 2020 Aug 13;10:13698. doi: 10.1038/s41598-020-70495-z (PMC7426858; doi:10.1038/s41598-020-70495-z)
Supplement: Supplementary file 8 — Supplementary Information 2. [file 41598_2020_70495_MOESM8_ESM.rtf]

Plot()
 {
 Sequence("North Pontic")
  {
   Boundary("North Pontic start");
   Phase("North Pontic samples")
   {
    R_Date("Dikiy Sad Poz-103214",2905,35)
    {
     latitude=46.980622;
     longitude=31.983558;
     color="red";
    };
    R_Date("Dikiy Sad Poz-105274",2895,35)
    {
     latitude=46.980622;
     longitude=31.983558;
     color="red";
    };
    R_Date("Guamsky Grot GrA-65338",2835,35)
    {
     latitude=44.224217;
     longitude=39.914883;
     color="red";
    };
    R_Date("Guamsky Grot Le-4235",2670,120)
    {
     latitude=44.224217;
     longitude=39.914883;
     color="red";
    };
    R_Date("Ivane-Puste Poz-103286",2490,30)
    {
     latitude=48.642218;
     longitude=26.187286;
     color="red";
    };
    R_Date("Ivane-Puste Poz-105276",2495,30)
    {
     latitude=48.642218;
     longitude=26.187286;
     color="red";
    };
    R_Date("Olbia Poz-105012",2020,30)
    {
     latitude=46.691388;
     longitude=31.904593;
     color="red";
    };
    R_Date("Rykan Poz-61889",2165,35)
    {
     latitude=51.621;
     longitude=39.552;
     color="red";
    }
    R_Date("Vinogradnyi Sad KIA-53658",3090,35)
    {
     latitude=47.759063;
     longitude=31.173591;
     color="red";
    };
    R_Date("Vinogradnyi Sad Poz-103215",3160,30)
    {
     latitude=47.759063;
     longitude=31.173591;
     color="red";
    };
    R_Date("Vinogradnyi Sad Poz-105275",3275,35)
    {
     latitude=47.759063;
     longitude=31.173591;
     color="red";
    };
    R_Date("Zalissya Poz-103216",2475,35)
    {
     latitude=48.665708;
     longitude=26.211781;
     color="red";
    };
    R_Date("Zanovskoe OxA-18316",2275,45)
    {
     latitude=48.826806;
     longitude=38.597556;
     color="red";
    };
    R_Date("Zanovskoe OxA-18317",2227,32)
    {
     latitude=48.826806;
     longitude=38.597556;
     color="red";
    };
    KDE_Plot("North Pontic dates")
    {
    };
   };
   Tau_Boundary("North Pontic end")
   {
   };
  };
  Sequence("Carpathian Basin")
  {
   Boundary("Carpathian Basin start");
   Phase("Carpathian Basin samples")
   {
    R_Date("Bosut Poz-98017",2680,30)
    {
     latitude=45.0893;
     longitude=19.1482;
     color="yellow";
    };
    R_Date("Cornești MAMS-35587",3048,18)
    {
     latitude=45.9;
     longitude=21.21666667;
     color="yellow";
    };
    R_Date("Cornești MAMS-35958",3085,21)
    {
     latitude=45.9;
     longitude=21.21666667;
     color="yellow";
    };
    R_Date("Cornești MAMS-35961",3087,22)
    {
     latitude=45.9;
     longitude=21.21666667;
     color="yellow";
    };
    R_Date("Cornești MAMS-35963",3068,23)
    {
     latitude=45.9;
     longitude=21.21666667;
     color="yellow";
    };
    R_Date("Cornești MAMS-35964",3049,22)
    {
     latitude=45.9;
     longitude=21.21666667;
     color="yellow";
    };
    R_Date("Cornești MAMS-35966",3051,32)
    {
     latitude=45.9;
     longitude=21.21666667;
     color="yellow";
    };
    R_Date("Crišnjevi Poz-104900",3120,70)
    {
     latitude=45.15556944;
     longitude=17.34666667;
     color="yellow";
    };
    R_Date("Dragomelj Poz-104925",2875,35)
    {
     latitude=46.109166;
     longitude=14.591111;
     color="yellow";
    };
    R_Date("Dragomelj Poz-104926",2855,35)
    {
     latitude=46.109166;
     longitude=14.591111;
     color="yellow";
    };
    R_Date("Dragomelj Poz-104927",2880,30)
    {
     latitude=46.109166;
     longitude=14.591111;
     color="yellow";
    };
    R_Date("Fajsz 18 OxA-26703",3075,36)
    {
     latitude=46.412545;
     longitude=18.919612;
     color="yellow";
    };
    R_Date("Fajsz 18 OxA-26704",3214,36)
    {
     latitude=46.412545;
     longitude=18.919612;
     color="yellow";
    };
    R_Date("Gasteil Poz-106219",2710,35)
    {
     latitude=47.7;
     longitude=15.9167;
     color="yellow";
    };
    R_Date("Gomolava Poz-98011",2240,30)
    {
     latitude=44.888333;
     longitude=19.748333;
     color="yellow";
    };
    R_Date("Gomolava Poz-98013",2630,30)
    {
     latitude=44.888333;
     longitude=19.748333;
     color="yellow";
    };
    R_Date("Gomolava Poz-98014",2525,30)
    {
     latitude=44.888333;
     longitude=19.748333;
     color="yellow";
    };
    R_Date("Gomolava Poz-98015",2670,30)
    {
     latitude=44.888333;
     longitude=19.748333;
     color="yellow";
    };
    R_Date("Gomolava Poz-98016",2775,30)
    {
     latitude=44.888333;
     longitude=19.748333;
     color="yellow";
    };
    R_Date("Kalakaèa Poz-100052",2725,30)
    {
     latitude=45.164722;
     longitude=20.080555;
     color="yellow";
    };
    R_Date("Lasinja Poz-104923",2115,30)
    {
     latitude=45.52861;
     longitude=15.8527;
     color="yellow";
    };
    R_Date("Mãgura-Buduiasca OxA-26706",3093,35)
    {
     latitude=45.529627;
     longitude=25.297481;
     color="yellow";
    };
    R_Date("Miercurea-Sibiului Poz-98086",2180,30)
    {
     latitude=45.85;
     longitude=22.833333;
     color="yellow";
    };
    R_Date("Neudorf Poz-106220",3045,35)
    {
     latitude=47.15;
     longitude=15.65;
     color="yellow";
    };
    R_Date("Orehova Vas Poz-104924",2865,35)
    {
     latitude=46.471666;
     longitude=15.666111;
     color="yellow";
    };
    R_Date("Orehova Vas Poz-104985",2870,40)
    {
     latitude=46.471666;
     longitude=15.666111;
     color="yellow";
    };
    R_Date("Oštrovi Poz-104921",3055,35)
    {
     latitude=45.152;
     longitude=17.34361111;
     color="yellow";
    };
    R_Date("Pécel 02 KIA-53627",3140,28)
    {
     latitude=47.49055;
     longitude=19.340833;
     color="yellow";
    };
    R_Date("Pécel 02 Poz-100051",2965,30)
    {
     latitude=47.49055;
     longitude=19.340833;
     color="yellow";
    };
    R_Date("Pécel 02 KIA-53628",3070,35)
    {
     latitude=47.49055;
     longitude=19.340833;
     color="yellow";
    };
    R_Date("Retznei Poz-106222",3095,35)
    {
     latitude=46.7372;
     longitude=15.5694;
     color="yellow";
    };
    R_Date("Szazhallombatta Poz-98088",2770,30)
    {
     latitude=47.300416;
     longitude=18.91361;
     color="yellow";
    };
    R_Date("Szazhallombatta Poz-98089",2075,30)
    {
     latitude=47.300416;
     longitude=18.91361;
     color="yellow";
    };
    R_Date("Teleac Poz-102424",2755,30)
    {
     latitude=46.06667;
     longitude=23.58333;
     color="yellow";
    };
    R_Date("Tribuna Poz-104928",2050,30)
    {
     latitude=46.055;
     longitude=14.50833;
     color="yellow";
    };
    R_Date("Tribuna Poz-104929",2455,30)
    {
     latitude=46.055;
     longitude=14.50833;
     color="yellow";
    };
    R_Date("Tribuna Poz-104971",2115,30)
    {
     latitude=46.055;
     longitude=14.50833;
     color="yellow";
    };
    R_Date("Tribuna Poz-104972",2145,30)
    {
     latitude=46.055;
     longitude=14.50833;
     color="yellow";
    };
    R_Date("Tribuna Poz-105051",2110,35)
    {
     latitude=46.055;
     longitude=14.50833;
     color="yellow";
    };
    R_Date("Vráble Poz-106228",3115,35)
    {
     latitude=48.2238;
     longitude=18.3135;
     color="yellow";
    };
    KDE_Plot("Carpathian Basin dates")
    {
    };
   };
   Tau_Boundary("Carpathian Basin end")
   {
   };
  };
  Sequence("Po Basin")
  {
   Boundary("Po Basin start");
   Phase("Po Basin samples")
   {
    R_Date("Custoza Poz-98009",3020,35)
    {
     latitude=45.371944;
     longitude=10.793333;
     color="violet";
    };
    R_Date("Lavagnone KIA-53633",3137,26)
    {
     latitude=45.43694;
     longitude=10.538055;
     color="violet";
    };
    R_Date("Lavagnone KIA-53634",3208,26)
    {
     latitude=45.43694;
     longitude=10.538055;
     color="violet";
    };
    R_Date("Lavagnone Poz-103897",3145,35)
    {
     latitude=45.43694;
     longitude=10.538055;
     color="violet";
    };
    R_Date("Santa Giulia Poz-107451",3065,30)
    {
     latitude=44.357734;
     longitude=10.609189;
     color="violet";
    };
    R_Date("Santa Giulia Poz-107452",3085,30)
    {
     latitude=44.357734;
     longitude=10.609189;
     color="violet";
    };
    R_Date("Lavagnone Poz-103898",3420,35)
    {
     Outlier();
     latitude=45.43694;
     longitude=10.538055;
     color="violet";
    };
    KDE_Plot("Po Basin dates")
    {
    };
   };
   Tau_Boundary("Po Basin end")
   {
   };
  };
  Sequence("central Europe")
  {
   Boundary("central Europe start");
   Phase("central Europe samples")
   {
    R_Date("Ansfelden UBA-39596",2303,29)
    {
     latitude=48.208333;
     longitude=14.288889;
     color="turquoise";
    };
    R_Date("Ansfelden UBA-39597",2293,33)
    {
     latitude=48.208333;
     longitude=14.288889;
     color="turquoise";
    };
    R_Date("Ansfelden BE-10047.1.1",2491,33)
    {
     latitude=48.208333;
     longitude=14.288889;
     color="turquoise";
    };
    R_Date("Binningen Poz-100056",2945,35)
    {
     latitude=47.5425;
     longitude=7.58;
     color="turquoise";
    };
    R_Date("Bruchenbrücken OxA-26700",3163,33)
    {
     latitude=50.329513;
     longitude=8.788007;
     color="turquoise";
    };
    R_Date("Fechenheim OxA-26701",2815,32)
    {
     latitude=50.130293;
     longitude=8.757341;
     color="turquoise";
    };
    R_Date("Goddelau OxA-26702",2484,34)
    {
     latitude=49.834846;
     longitude=8.493193;
     color="turquoise";
    };
    R_Date("Hagnau Poz-106140",2780,35)
    {
     latitude=47.673584;
     longitude=9.321719;
     color="turquoise";
    };
    R_Date("Ipf KIA-53631",2818,27)
    {
     latitude=48.870222;
     longitude=10.356771;
     color="turquoise";
    };
    R_Date("Knittlingen Poz-106225",2785,35)
    {
     latitude=49.027667;
     longitude=8.760693;
     color="turquoise";
    };
    R_Date("Königshofen Poz-106223",3060,35)
    {
     latitude=49.559429;
     longitude=9.704704;
     color="turquoise";
    };
    R_Date("Königshofen Poz-106224",3020,35)
    {
     latitude=49.559429;
     longitude=9.704704;
     color="turquoise";
    };
    R_Date("Lipnik 5 Poz-103203",3040,35)
    {
     latitude=49.993333;
     longitude=22.311388;
     color="turquoise";
    };
    R_Date("Lipnik 5 Poz-104894",3025,30)
    {
     latitude=49.993333;
     longitude=22.311388;
     color="turquoise";
    };
    R_Date("Maszkowice Poz-104888",3040,30)
    {
     latitude=49.554444;
     longitude=20.465277;
     color="turquoise";
    };
    R_Date("Maszkowice Poz-104889",2465,35)
    {
     latitude=49.554444;
     longitude=20.465277;
     color="turquoise";
    };
    R_Date("Maszkowice Poz-104890",2520,30)
    {
     latitude=49.554444;
     longitude=20.465277;
     color="turquoise";
    };
    R_Date("Maszkowice Poz-104893",2935,30)
    {
     latitude=49.554444;
     longitude=20.465277;
     color="turquoise";
    };
    R_Date("Meidling Poz-102432",2525,30)
    {
     latitude=48.32694;
     longitude=15.6225;
     color="turquoise";
    };
    R_Date("Meidling Poz-102433",2495,30)
    {
     latitude=48.32694;
     longitude=15.6225;
     color="turquoise";
    };
    R_Date("Meidling Poz-102434",2545,35)
    {
     latitude=48.32694;
     longitude=15.6225;
     color="turquoise";
    };
    R_Date("Meidling Poz-102436",2490,30)
    {
     latitude=48.32694;
     longitude=15.6225;
     color="turquoise";
    };
    R_Date("Pisek-Sever Poz-102431",3065,35)
    {
     latitude=49.33027;
     longitude=14.11166;
     color="turquoise";
    };
    R_Date("Soví pøevis Poz-102422",3055,35)
    {
     latitude=50.5225;
     longitude=15.0772;
     color="turquoise";
    };
    R_Date("Stillfried Poz-103902",2810,30)
    {
     latitude=48.41416;
     longitude=16.8375;
     color="turquoise";
    };
    R_Date("Stillfried Poz-103903",2805,35)
    {
     latitude=48.41416;
     longitude=16.8375;
     color="turquoise";
    };
    R_Date("Stillfried Poz-103904",2835,35)
    {
     latitude=48.41416;
     longitude=16.8375;
     color="turquoise";
    };
    R_Date("Valeèov 1 Poz-102423",2940,35)
    {
     latitude=50.5083;
     longitude=15.02916;
     color="turquoise";
    };
    R_Date("Velim GrN-27618",3126,20)
    {
     latitude=50.066667;
     longitude=15.1;
     color="turquoise";
    };
    R_Date("Witów Poz-104887",2990,35)
    {
     latitude=50.149722;
     longitude=20.5825;
     color="turquoise";
    };
    R_Date("Zahájí Poz-29572",3140,40)
    {
     latitude=50.37888889;
     longitude=14.11777778;
     color="turquoise";
    };
    R_Date("Ipf Poz-106789",3580,35)
    {
     Outlier();
     latitude=48.870222;
     longitude=10.356771;
     color="turquoise";
    };
    KDE_Plot("central Europe dates")
    {
    };
   };
   Tau_Boundary("central Europe end")
   {
   };
  };
  Sequence("North-central Europe")
  {
   Boundary("North-central Europe start");
   Phase("North-central Europe samples")
   {
    R_Date("Altenrheine Poz-107448",2915,30)
    {
     latitude=52.300265;
     longitude=7.480408;
     color="pink";
    };
    R_Date("Badegow KIA-53023",2905,50)
    {
     latitude=53.573091;
     longitude=11.732475;
     color="pink";
    };
    R_Date("Badegow KIA-53024",2990,35)
    {
     latitude=53.573091;
     longitude=11.732475;
     color="pink";
    };
    R_Date("Borgstedt Poz-111228",2404,32)
    {
     latitude=54.333567;
     longitude=9.703932;
     color="pink";
    };
    R_Date("Borken Poz-107449",2390,30)
    {
     latitude=51.843897;
     longitude=6.862341;
     color="pink";
    };
    R_Date("Brekendorf Poz-111227",2683,31)
    {
     latitude=54.423512;
     longitude=9.629328;
     color="pink";
    };
    R_Date("Butzow Poz-111229",2837,33)
    {
     latitude=53.834805;
     longitude=13.643222;
     color="pink";
    };
    R_Date("Depenau 17 Poz-97634",2770,30)
    {
     latitude=54.161673;
     longitude=10.212455;
     color="pink";
    };
    R_Date("Großbahren Poz-102427",2965,35)
    {
     latitude=51.715083;
     longitude=13.683342;
     color="pink";
    };
    R_Date("Hittfeld Poz-97511",2410,30)
    {
     latitude=53.392316;
     longitude=10.004316;
     color="pink";
    };
    R_Date("Hundisburg KIA-33853",2080,30)
    {
     latitude=52.244444;
     longitude=11.397778;
     color="pink";
    };
    R_Date("Jülich-Güsten Poz-107450",2885,30)
    {
     latitude=50.956251;
     longitude=6.429092;
     color="pink";
    };
    R_Date("Lüdelsen 6 KIA42499",2685,40)
    {
     latitude=52.68689;
     longitude=10.941398;
     color="pink";
    };
    R_Date("Lüdelsen 6 KIA42500",2785,35)
    {
     latitude=52.68689;
     longitude=10.941398;
     color="pink";
    };
    R_Date("Lutomiersk Poz-43498",2755,30)
    {
     latitude=51.7525;
     longitude=19.225;
     color="pink";
    };
    R_Date("Lutomiersk Poz-43504",2810,35)
    {
     latitude=51.7525;
     longitude=19.225;
     color="pink";
    };
    R_Date("Lutomiersk Poz-104897",2760,35)
    {
     latitude=51.7525;
     longitude=19.225;
     color="pink";
    };
    R_Date("Möthlow Poz-102426",2880,35)
    {
     latitude=52.600314;
     longitude=12.652357;
     color="pink";
    };
    R_Date("Olbetal Poz-97512",2805,30)
    {
     latitude=52.245736;
     longitude=11.389689;
     color="pink";
    };
    R_Date("Olbetal Poz-97513",2905,30)
    {
     latitude=52.245736;
     longitude=11.389689;
     color="pink";
    };
    R_Date("Olbetal Poz-97593",2890,30)
    {
     latitude=52.245736;
     longitude=11.389689;
     color="pink";
    };
    R_Date("Olbetal Poz-97594",2890,30)
    {
     latitude=52.245736;
     longitude=11.389689;
     color="pink";
    };
    R_Date("Olbetal Poz-97595",2905,30)
    {
     latitude=52.245736;
     longitude=11.389689;
     color="pink";
    };
    R_Date("Pasewalk Poz-111230",2947,32)
    {
     latitude=53.491058;
     longitude=13.994479;
     color="pink";
    };
    R_Date("Radis KIA-38121",2994,36)
    {
     latitude=51.75;
     longitude=12.516667;
     color="pink";
    };
    R_Date("Rullstorf KIA-9544",2963,29)
    {
     latitude=53.291117;
     longitude=10.516751;
     color="pink";
    };
    R_Date("Rullstorf Poz-103615",2955,35)
    {
     latitude=53.291117;
     longitude=10.516751;
     color="pink";
    };
    R_Date("Schwerin Poz-97596",2945,30)
    {
     latitude=53.624793;
     longitude=11.404171;
     color="pink";
    };
    R_Date("Smuszewo 3 Poz-103288",2488,30)
    {
     latitude=52.88333333;
     longitude=17.41666667;
     color="pink";
    };
    R_Date("Szczepidlo 17 Poz#2-108754",3000,29)
    {
     latitude=52.21666667;
     longitude=18.33333333;
     color="pink";
    };
    R_Date("Vogelsang Poz-97597",2325,30)
    {
     latitude=53.747112;
     longitude=12.408392;
     color="pink";
    };
    R_Date("Vogelsang Poz-97623",2605,30)
    {
     latitude=53.747112;
     longitude=12.408392;
     color="pink";
    };
    R_Date("Wahlstedt Poz-111231",2865,35)
    {
     latitude=53.962827;
     longitude=10.209475;
     color="pink";
    };
    R_Date("Walsleben KIA-33855",2182,33)
    {
     latitude=52.759722;
     longitude=11.849722;
     color="pink";
    };
    R_Date("Warendorf Poz-107447",2875,30)
    {
     latitude=51.973787;
     longitude=7.902394;
     color="pink";
    };
    R_Date("Watenstedt Poz-100054",2835,30)
    {
     latitude=52.088589;
     longitude=10.836536;
     color="pink";
    };
    R_Date("Watenstedt Poz-100055",2795,35)
    {
     latitude=52.088589;
     longitude=10.836536;
     color="pink";
    };
    R_Date("Wismar Poz-103900",2590,35)
    {
     latitude=53.896146;
     longitude=11.469083;
     color="pink";
    };
    R_Date("Wismar Poz-103901",2535,35)
    {
     latitude=53.896146;
     longitude=11.469083;
     color="pink";
    };
    R_Date("Zweedorf Poz-97624",2515,30)
    {
     latitude=54.079829;
     longitude=11.673373;
     color="pink";
    };
    KDE_Plot("North-central Europe dates")
    {
    };
   };
   Tau_Boundary("North-central Europe end")
   {
   };
  };
  Sequence("South-east Europe")
  {
   Boundary("South-east Europe start");
   Phase("South-east Europe samples")
   {
    First("South-east Europe first");
    R_Date("Assiros HD-25509",3071,12)
    {
     latitude=40.820833;
     longitude=23.030833;
     color="black";
    };
    R_Date("Hisar Poz-105052",2965,35)
    {
     latitude=42.991388;
     longitude=21.9375;
     color="black";
    };
    R_Date("Hisar Poz-98085",2920,35)
    {
     latitude=42.991388;
     longitude=21.9375;
     color="black";
    };
    KDE_Plot("South-east Europe dates")
    {
    };
   };
   Tau_Boundary("South-east Europe end")
   {
   };
  };
    Page( );
  Phase("time lags")
  {
   Difference("North Pontic before Carpathian Basin", "Carpathian Basin start", "North Pontic start");
   Difference("Carpathian Basin before Po Basin", "Po Basin start", "Carpathian Basin start");
   Difference("Carpathian Basin before central Europe", "central Europe start", "Carpathian Basin start");
   Difference("central Europe before  North-central Europe", "North-central Europe start", "central Europe start");
   Difference("Carpathian Basin before South-east Europe", "South-east Europe start", "Carpathian Basin start");
  };
Page( );
  Sequence("date of the Tollense battle")
  {
   Boundary("start");
   Phase("radiocarbon samples regarded as dating to the conflict horizon")
   {
    Phase("results reported in Jantzen et al (2011)")
    {
     R_Date("bone Wa IV/89/654 AAR-11146", 2980, 38);
     R_Date("bone Wa IV/85/311,1 AAR-11150", 2952, 40);
     R_Date("bone 2000/1382,2 AAR-11148", 2989, 33);
     R_Date("bone 2000/1382,3 AAR-11149", 2982, 38);
     R_Date("bone 2009/1004 KIA-40076", 2980, 25);
     R_Date("bone 1996/855,8 AAR-11151", 2992, 39);
     R_Date("bone 1996/855,281 AAR-11152", 2957, 34);
     R_Date("bone 1996/855,99 AAR-11153", 2888, 39);
     R_Date("bone KN-5020", 3079, 54)
     {
      Outlier();
     };
     R_Date("wooden club UtC-9740", 3070, 50)
     {
      Outlier();
     };
     R_Date("bone 1996/855,927 AAR-11154", 2945, 38);
     R_Date("bone Wa IV/78/225 AAR-11147", 2958, 32);
    };
    Phase("additional results reported in Jantzen et al (2017)")
    {
     R_Date("bone Wa IV/85/311,9 AAR-17097", 2983, 29);
     R_Date("human tooth 2000/1382,1 AAR-17098", 2864, 32)
     {
      Outlier();
     };
     R_Date("horse tooth 2014/1231,7 AAR-21703", 2977, 25);
    };
    Phase("additional results reported in Terberger et al (2018)")
    {
     R_Date("skull AAR-19796", 2977,29);
     R_Date("dogwood arrow AAR-19794", 3017,28);
    };
    Phase("additional results reported in Uhlig et al (2019)")
    {
     R_Date("birch awl handle MAMS-29473", 2988, 24);
     R_Date("dogwood arrow MAMS-29471", 3010, 25);
     R_Date("dogwood arrow Poz-84545", 3055, 35);
     R_Date("second arrow MAMS-29472", 3038, 29);
    };
    Span("constrained", N(20,10));//assumes that wood-age offsets and collagen turnover times are normally distributed with a mean of 20+-10 years
    Last("battle date");//the last-forming sample is assumed to be contemporaneous with the battle itself
   };
   Boundary("end");
  };
  Line( );
  Phase("dendrochronology of oak timbers potentially associated with the battle (reported in Jantzen et al 2017)")
  {
   C_Date("2008 oak pile, sapwood not reported", -1301,0);
   C_Date("2012 oak pile, waney edge (felled 1320 BC)", -1320,0);
   Combine("east bank structure")//the structure is assumed to have been built with freshly-felled timbers, which must therefore be identical in date
   {
    C_Date("timber no2 (felled 1225+-10 BC)", -1225, 10);
    C_Date("timber no3 (felled 1218+-10 BC)", -1218, 10);
    C_Date("timber no4 (felled 1228+-10 BC)", -1228, 10);
   };
  };
Phase()
  {
   Date("=North-central Europe start");
   Date("=battle date");
  };
 };
