# Supplementary material for: New AMS 14C dates track the arrival and spread of broomcorn millet cultivation and agricultural change in prehistoric Europe
Source: Sci Rep. 2020 Aug 13;10:13698. doi: 10.1038/s41598-020-70495-z (PMC7426858; doi:10.1038/s41598-020-70495-z)
Supplement: Supplementary file 9 — Supplementary Information 3. [file 41598_2020_70495_MOESM9_ESM.pdf]

Submission form for millet ^{14}C dating programme

Dragana Filipović (d.filipovic@ufg.uni-kiel.de)
Marta Dal Corso (mdalcorso@gshdl.uni-kiel-de)
Institute for Pre- and Protohistory
Kiel University
Johanna-Mestorf Str. 2-6, 24114 Kiel, Germany

SCALES OF TRANSFORMATION

Family: Poaceae   Genus: Species:

Country: Name of site, location (e.g. nearest village/town, county):

Latitude: ° ' " N   Longitude: ° ' " E

Type of material:	Number of items
Grain (karyopsis)	
'Clump' of fused grains	

SENDER

Name, surname, title:

Address:

Tel: Email: Submission date (D/M/Y): / /

SAMPLE PROVENANCE

Original sample number (e.g. flotation sample):

Excavation layer: Feature number:

Quadrant: Trench number:

Sector: Other:

1. Chronological period:

2. Archaeological culture:

3. Type of archaeological site (please tick all relevant):

Settlement ☐ Cave ☐ Pile dwellings ☐ Cemetery ☐ Tell ☐ Multi-period site ☐

Ritual site ☐ Flat settlement ☐ Single-phase site ☐ Other:

4. Local geological/environmental unit in which the site is located (e.g. alluvial plain):

5. Archaeological context from which the grain(s) were recovered (please mark all relevant):

House ☐ Posthole ☐ Grave ☐ Occupation layer ☐ Oven ☐ Daub ☐ Pit ☐ Hearth ☐

Pot content ☐ Midden ☐ Basin ☐ Bin ☐ Ditch ☐ Other:

6. How many millet grains were found in the context (i.e. few or a concentration)? Were they scattered through the context or were they found together within a discrete area (e.g. within a lens of charred material in a pit)?

7.How were the grains collected (e.g. hand-picked, dry/wet sieving, manual or machine flotation)?

DFG
CAU Kiel University Christian-Albrechts-Universität zu Kiel
LANDESMUSEEN GOTTORF
JMA
KIEL archaeology

Dragana Filipović (d.filipovic@ufg.uni-kiel.de)
Marta Dal Corso (mdalcorso@gshdl.uni-kiel-de)
Institute for Pre- and Protohistory
Kiel University
Johanna-Mestorf Str. 2-6, 24114 Kiel, Germany

8.How big was the sample in which the grains were found (i.e. volume/weight of flotation sample)? If you can, please provide the number of millet grains per litre (or kg) of sediment (i.e. density).

9.How were the sample or the grain(s) stored (e.g. air-dried and placed in a plastic/paper bag or plastic vial/glass tube or aluminium foil)? Were any preservatives, fungicides, etc. used?

10. Are you aware of any possible contamination of the material or the context (please mark all relevant):

Modern vegetation ☐ Volcanic environment ☐ Animal intrusions ☐ Calcareous environment ☐
Physical disturbance (e.g. ploughing) ☐ Nearness to water table ☐ Carbonates ☐ Nearness to surface ☐
Humic acids ☐ Other:

11. Are/were these or any other related seeds selected for dating within a different project?

12. Was any material/object from the same context absolute-dated? Please provide the reference and the date if known and published:

13. Please list any relevant publications that can help us contextualise the results:

14. Additional information:

SCALES OF TRANSFORMATION
